# Dichotomy between Regulation of Coral Bacterial Communities and Calcification Physiology under Ocean Acidification Conditions

**DOI:** 10.1128/AEM.02189-20

**Published:** 2021-02-26

**Authors:** A. Shore, R. D. Day, J. A. Stewart, C. A. Burge

**Affiliations:** a Institute of Marine and Environmental Technology, University of Maryland Baltimore County, Baltimore, Maryland, USA; b Department of Biology, Farmingdale State College, Farmingdale, New York, USA; c Marine Science and Nautical Training Academy, Charleston, South Carolina, USA; d Hollings Marine Laboratory, National Institute of Standards and Technology, Charleston, South Carolina, USA; e School of Earth Science, University of Bristol, Bristol, United Kingdom; University of Illinois at Chicago

**Keywords:** ocean acidification, bacterial community, coral, microbiome, calcification, trace elements, boron isotopes, Maug Caldera, CO_2_ seep, *Endozoicomonas*, coral microbiome

## Abstract

Ocean acidification (OA) threatens the growth and function of coral reef ecosystems. A key component to coral health is the microbiome, but little is known about the impact of OA on coral microbiomes. A submarine CO_2_ vent at Maug Island in the Northern Mariana Islands provides a natural pH gradient to investigate coral responses to long-term OA conditions. Three coral species (*Pocillopora eydouxi*, *Porites lobata*, and *Porites rus*) were sampled from three sites where the mean seawater pH is 8.04, 7.98, and 7.94. We characterized coral bacterial communities (using 16S rRNA gene sequencing) and determined pH of the extracellular calcifying fluid (ECF) (using skeletal boron isotopes) across the seawater pH gradient. Bacterial communities of both *Porites* species stabilized (decreases in community dispersion) with decreased seawater pH, coupled with large increases in the abundance of *Endozoicomonas*, an endosymbiont. *P. lobata* experienced a significant decrease in ECF pH near the vent, whereas *P. rus* experienced a trending decrease in ECF pH near the vent. In contrast, *Pocillopora* exhibited bacterial community destabilization (increases in community dispersion), with significant decreases in *Endozoicomonas* abundance, while its ECF pH remained unchanged across the pH gradient. Our study shows that OA has multiple consequences on *Endozoicomonas* abundance and suggests that *Endozoicomonas* abundance may be an indicator of coral response to OA. We reveal an interesting dichotomy between two facets of coral physiology (regulation of bacterial communities and regulation of calcification), highlighting the importance of multidisciplinary approaches to understanding coral health and function in a changing ocean.

**IMPORTANCE** Ocean acidification (OA) is a consequence of anthropogenic CO_2_ emissions that is negatively impacting marine ecosystems such as coral reefs. OA affects many aspects of coral physiology, including growth (i.e., calcification) and disrupting associated bacterial communities. Coral-associated bacteria are important for host health, but it remains unclear how coral-associated bacterial communities will respond to future OA conditions. We document changes in coral-associated bacterial communities and changes to calcification physiology with long-term exposure to decreases in seawater pH that are environmentally relevant under midrange IPCC emission scenarios (0.1 pH units). We also find species-specific responses that may reflect different responses to long-term OA. In *Pocillopora*, calcification physiology was highly regulated despite changing seawater conditions. In *Porites* spp., changes in bacterial communities do not reflect a breakdown of coral-bacterial symbiosis. Insights into calcification and host-microbe interactions are critical to predicting the health and function of different coral taxa to future OA conditions.

## Abstract (precis)

Ocean acidification (OA) is a consequence of anthropogenic CO_2_ emissions that is negatively impacting marine ecosystems such as coral reefs. OA affects many aspects of coral physiology, including growth (i.e., calcification) and disrupting associated bacterial communities.

## INTRODUCTION

Global climate change is altering the structure and function of marine ecosystems worldwide (1). Increases in seawater temperature are changing the distribution of suitable habitat (2), increasing disease outbreaks (3, 4), and contributing to population and productivity decline (5, 6). Coral reef ecosystems are considered particularly vulnerable, having recently experienced several thermally induced mass-bleaching events (the breakdown in symbiosis with intercellular algae, family *Symbiodiniaceae*) (7). Ocean acidification (OA) is another rapidly emerging consequence of anthropogenic carbon emissions that is negatively impacting marine ecosystems (6). It is estimated that more than a quarter of CO_2_ emissions are taken up by the ocean (8), leading to OA or the reduction of seawater pH and calcium carbonate saturation states in marine environments (9). Ocean pH has already decreased by ∼0.1 pH units since the beginning of the industrial revolution and is expected to decrease by another 0.2 to 0.4 pH units by 2100 (10). OA threatens the growth and persistence of many calcifying organisms, including calcareous phytoplankton, pteropods, shellfish, and scleractinian corals (6, 11). Given the cultural, economic, and ecological importance of tropical coral reef ecosystems, there is a pressing need to predict the physiological response of corals to future ocean changes.

OA effects on coral growth are well-understood; decreased calcification rates or skeletal density and facilitated bioerosion in many coral species can lead to net reef dissolution (12). Some corals appear more resistant to OA than others (13–15), yet it remains unclear how such acclimatization/adaptation is achieved (16). Many scleractinian corals optimize conditions for calcification by transporting Ca^2+^ from seawater into their extracellular calcifying fluid (ECF) in exchange for H^+^ ions via the Ca-ATPase pump (17). It has been suggested that this “pH upregulation” of the ECF relative to ambient seawater is key to calcification under OA conditions, yet coral taxa exhibit a wide range of capabilities for modifying internal seawater carbonate chemistry (18–20). The extent to which pH upregulation occurs on coral reefs currently impacted by low-pH conditions and which coral taxa are more resilient is not well understood.

OA also impacts many aspects of coral physiology, including reproduction (21), larval settlement (22, 23), juvenile development (24), symbiosis with *Symbiodiniaceae* (25) and associated microbiomes (26, 27). Stable, mutualistic microbiomes are important to coral health and to increased resilience to environmental perturbations (28, 29). Under environmental stress/change, microbial community dynamics have multiple potential responses: (i) hosts can retain homeostasis with their microbial communities despite environmental change (i.e., resistance/resilience), (ii) hosts can restructure microbial communities to adjust to new environmental conditions (i.e., acclimation), and (iii) environmental conditions may break down symbiosis between hosts and microbial communities (i.e., dysbiosis) (30). For example, short-term, experimental studies have shown that OA can destabilize coral-associated bacterial communities (31–33) and reduce the rate of microbially mediated nitrogen fixation (34), potentially predicting microbial dysbiosis for future coral reefs. On the other hand, some marine taxa, such as sponges, may modify the composition of associated bacterial communities to maintain its functional stability under OA, leading to higher survival rates under OA stress (35). However, these experimental OA studies often transplant hosts into low-pH conditions with little adaptation/acclimatization time and have limited experimental exposure to low-pH conditions, making them unrealistic models for understanding the response to future OA. Microbial community dynamics in benthic marine hosts are poorly documented in response to chronic, long-term low-pH conditions.

**FIG 1:**
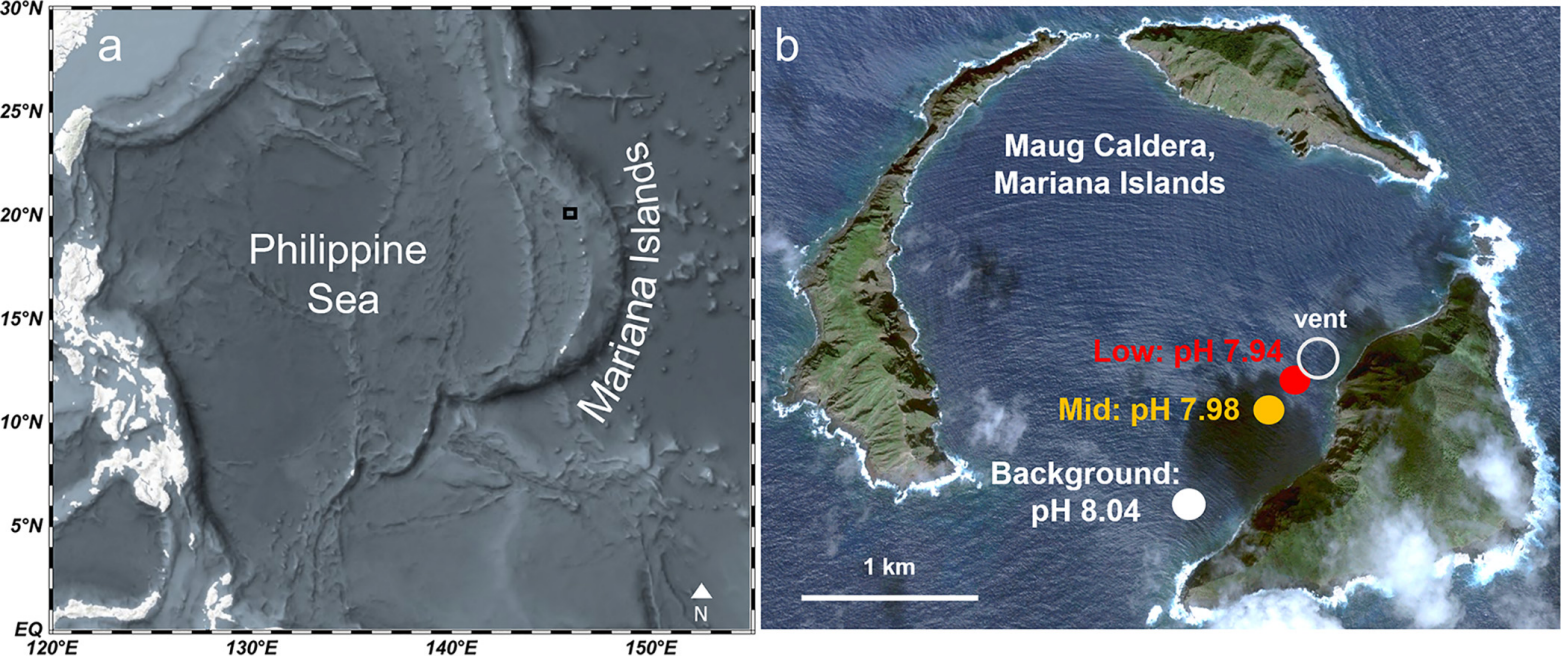
(a and b) Maug islands within the Mariana Islands (a) and Maug Caldera (b) with background-pH, mid-pH, and low-pH sites of coral collection. Panel b is based on a map from Google Earth (https://earth.google.com/web/@20.02245847,145.22423532,1.58796908a,5159.09405382d,35y,0h,0t,0r).

Shallow underwater volcanic vents provide unique natural laboratories to investigate coral reef health under long-term, low seawater pH. Maug Caldera (Northern Mariana Islands) (Fig. 1) provides one such example in which corals experience a gradient of pH ranging from average ambient surface seawater to OA conditions projected to occur within the next 50 years (10). The multifaceted responses of the coral host together with its *Symbiodiniaceae* and microbiome (referred to as the “coral holobiont”) to OA conditions requires multidisciplinary approaches. In this study, we used 16S rRNA gene amplicon sequencing and biogeochemistry approaches in three coral species (*Pocillopora eydouxi*, *Porites lobata*, and *Porites rus*) to examine (i) coral-associated bacterial community response (resistance, acclimation, or dysbiosis) and (ii) the ability of the coral host to upregulate internal pH with long-term exposure to low pH seawater.

## RESULTS

### Changes in bacterial communities across the pH gradient were coral species-specific.

Bacterial communities from *P. eydouxi*, *P. lobata*, and *P. rus* samples are referred to here as “Poc-communities,” “Plob-communities,” and “Prus-communities,” respectively. After quality filtering, 6,097,305 high-quality sequences were obtained from all coral samples analyzed (*n* = 82) (Table 1). After the removal of mitochondrial, chloroplast, and unassigned reads, the total pool of sequences were assigned to 3,918 amplicon sequence variants (ASVs). All samples clustered into three distinct groups by coral species, but Plob-communities and Prus-communities were more similar to each other than either were to Poc-communities (see Fig. S1 in the supplemental material). Bacterial communities in each coral species were all significantly different from each other (analysis of similarities [ANOSIM]: *P* < 0.01; see Table S1 in the supplemental material). Thus, remaining analyses were conducted on each coral species separately.

**TABLE 1:** Summary description of samples, collection site seawater chemistry, and sequence analysis^*a*^

Species	*n*	Site^*b*^			Sequence analysis summary		
		Name	pH	pCO_2_ (μatm)	High-quality bacterial sequences^*c*^	Observed ASVs^*d*^	Shannon H′
*Pocillopora eydouxi*	10	Background pH	8.04 (0.016)	401.2 (4.61)	42,521 (4,251)	31 (3)^A^	3.71 (0.19)^A^
	9	Mid pH	7.98 (0.027)	441.2 (21.23)	28,556 (5,298)	27 (3)^A^	3.65 (0.22)^A^
	8	Low pH	7.94 (0.051)	502.0 (29.67)	9,205 (3,168)	21 (7)^A^	3.11 (0.43)^A^
*Porites lobata*	7	Background pH	8.04 (0.016)	401.2 (4.61)	4,202 (1,105)	71 (12)^A^	5.35 (0.19)^A^
	10	Mid pH	7.98 (0.027)	441.2 (21.23)	13,154 (2,677)	245 (33)^B^	7.02 (0.28)^B^
	10	Low pH	7.94 (0.051)	502.0 (29.67)	3,968 (598)	35 (15)^C^	3.50 (0.48)^C^
*Porites rus*	9	Background pH	8.04 (0.016)	401.2 (4.61)	6,036 (1,593)	33 (9)^A^	3.65 (0.25)^A^
	9	Mid pH	7.98 (0.027)	441.2 (21.23)	5,610 (1,078)	26 (3)^A^	3.55 (0.14)^A^
	10	Low pH	7.94 (0.051)	502.0 (29.67)	4,487 (1,468)	19 (4)^A^	3.02 (0.22)^A^

a Alpha-diversity data were determined from rarefied ASV tables and include metrics for richness (observed ASVs) and diversity (Shannon H′). Seawater chemistry data are presented as means (± standard deviations). All diversity data are presented as means (standard errors). Alpha-diversity metrics (within a species) were compared by using a nonparametric Kruskal-Wallis test with Mann-Whitey *post hoc* comparisons. Comparisons that do not share a superscript capital letter are significantly different at *P* ≤ 0.05.

b The seawater chemistry data (pH and pCO_2_) are from Enochs et al. (70).

c Quality filtering included removal of low-quality, short, mitochondrial, chloroplast, and unassigned reads.

d Amplicon sequence variants (ASVs) after rarifying to equal sequence sampling depth (1,100 reads).

**FIG 2:**
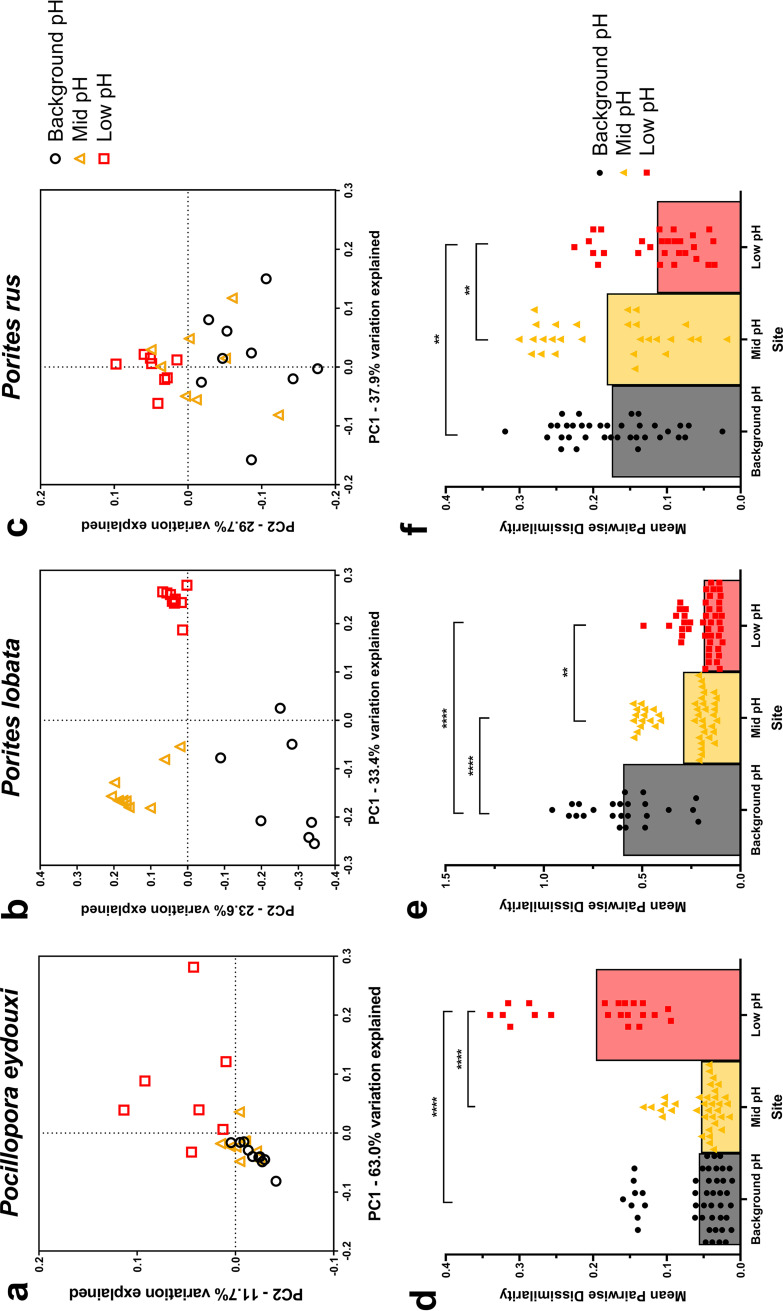
(a to c) PCoA plot of the weighted UniFrac distance matrices for *Pocillopora eydouxi* (a), *Porites lobata* (b), and *Porites rus* (c) bacterial communities. (d to f) Mean (with individual values) pairwise dissimilarity values for bacterial communities within a site for *Pocillopora eydouxi* (d), *Porites lobata* (e), and *Porites rus* (f). Black circles, background-pH site; gold triangles, mid-pH site; red squares, low-pH site. Kruskal-Wallis tests with Dunn’s multiple comparisons were performed across sites within a species. **, *P* < 0.01; ***, *P* < 0.001; ****, *P* < 0.0001.

Bacterial communities of all three coral species shifted significantly across the pH gradient, but in a species-specific manner (ANOSIM: *P* < 0.05, Fig. 2a to c, Table 2). Poc-communities from background-pH and mid-pH sites were highly similar with tight clustering of samples from these sites, but Poc-communities from the low-pH site dispersed, indicating changes in overall community structure near the vent (Fig. 2a; Table 2). Plob-communities distinctly clustered at each site (Fig. 2b) and were significantly different at each site (Table 2). Prus-communities experienced changes across the pH gradient with much overlap between sites (Figure 2c); however, a significant shift in Prus-community structure occurred at the low-pH site, but not between background-pH and mid-pH sites (Table 2).

**TABLE 2:** Pairwise ANOSIM comparisons of the weighted UniFRAC distance matrix values

Coral species	Sites compared	Global R	*P*
*Pocillopora eydouxi*	Background vs mid	0.073	0.125
	**Background vs low**	**0.493**	**0.001**
	**Mid vs low**	**0.425**	**0.010**
*Porites lobata*	**Background vs mid**	**0.595**	**0.001**
	**Background vs low**	**0.731**	**0.001**
	**Mid vs low**	**0.560**	**0.001**
*Porites rus*	Background vs mid	0.033	0.657
	**Background vs low**	**0.096**	**0.035**
	Mid vs low	0.037	0.146

a A Global R value of 0 means no differences in bacterial community structure, whereas a Global R value of 1 means complete differences in bacterial community structure. Comparisons with significant differences are indicated in boldface.

The species-specific responses to the pH gradient are more clearly visualized in the sample-to-sample variation, or community dispersion, in bacterial communities, represented by mean pairwise dissimilarity, within each site. Poc-communities destabilized across the pH gradient, with a significant increase in community dispersion occurring at the low-pH site (Fig. 2d). In contrast, Plob-communities stabilized across the pH gradient, with significant decreases in community dispersion occurring at both mid- and low-pH sites (Fig. 2e). Prus-communities also stabilized, but the decrease in community dispersion was at the low-pH site only (Fig. 2f).

Both richness (observed ASVs) and Shannon diversity were consistent in Poc-communities and in Prus-communities across the pH gradient. In Plob-communities, both the Shannon diversity and the ASV richness were impacted across the pH gradient (Shannon Diversity: Kruskal-Wallis; H = 17.25, *P* = 0.0002) (ASV richness: Kruskal-Wallis; H = 19.07, *P* = 0.0001) (Table 1). Richness and Shannon diversity were significantly higher at the mid-pH site, but at the low-pH site the Shannon diversity was significantly lower in Plob-communities. Plob-communities at the mid-pH site were distinct due to the presence of 1,940 ASVs (59.1% of the total Plob-community ASVs) that were unique to Plob-communities at the mid-pH site.

**FIG 3:**
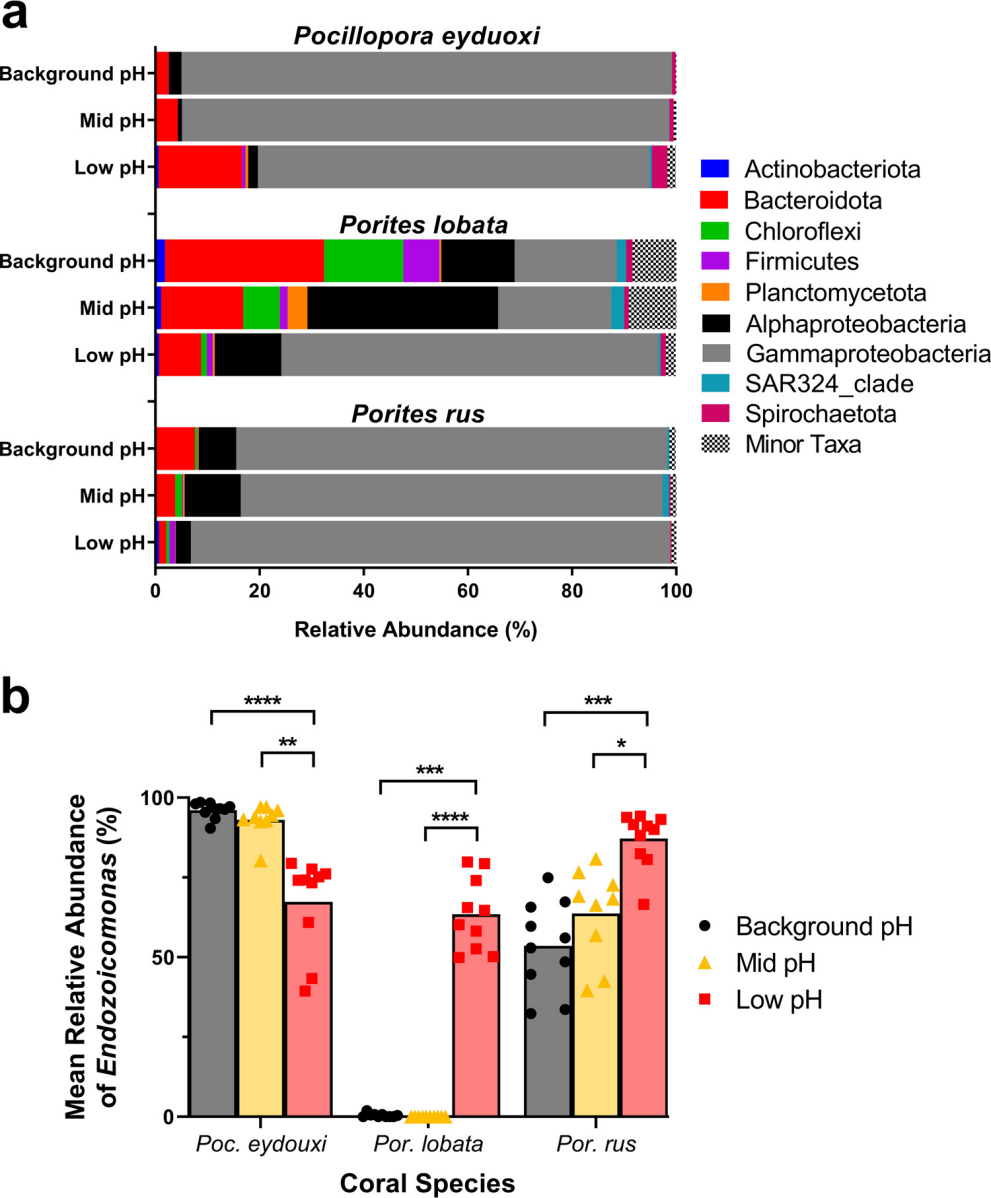
(a) Mean percent abundances of bacterial sequences for *Pocillopora eydouxi*, *Porites lobata*, and *Porites rus* coral samples at background-pH, mid-pH, and low-pH sites. Bacterial taxonomy was assigned to phyla or class using the Silva v138 database. All taxa of <1% of the total community were grouped under the category “minor taxa.” (b) Relative abundances of *Endozoicomonas* sequences in all three coral species across sites. Black, background-pH site; gold, mid-pH site; red, low-pH site. Data represent means with individual values. Kruskal-Wallis test with Dunn’s multiple comparisons were conducted across sites within a species. *, *P* < 0.05; **, *P* < 0.01; ***, *P* < 0.001; ****, *P* < 0.0001.

Proteobacteria was the most abundant phylum in all three species at all sites. In Poc-communities, *Gammaproteobacteria* averaged 94.1 ± 2.1% of the total community at the background-pH site, but this was replaced by significant increases in *Bacteroidota* and *Spirochaetota* at the low-pH site (Kruskal-Wallis: *P* < 0.02) (Fig. 3a). In Plob-communities, phyla were more evenly distributed at the Background pH site and were represented primarily by *Bacteroidota* (34.6 ± 7.5%), *Gammaproteobacteria* (19.6. ± 6.3%), *Chloroflexi* (15.2 ± 6.0%), *Alphaproteobacteria* (10.1 ± 2.7%), and *Firmicutes* (6.9 ± 5.4%) (Fig. 3a). In Plob-communities, *Alphaproteobacteria* and *Planctomycetota* increased significantly at the mid-pH site and *Gammaproteobacteria* increased significantly at the low-pH site (Kruskal-Wallis: *P* < 0.02) (Fig. 3a). Prus-communities were also dominated by *Gammaproteobacteria*, averaging 82.8 ± 5.5% of the total community at the background-pH site, and there were no significant changes in abundance of any major phyla in Prus-communities across the pH gradient (Kruskal-Wallis: *P* > 0.05) (Fig. 3a).

DESeq2 analysis identified 86 ASVs that were differentially abundant at either the low-pH or the mid-pH site, compared to the background-pH site (see Table S2). In Poc-communities, an ASV classified as “*Candidatus* Amoebophilus” was enriched at both mid-pH and low-pH sites, whereas five ASVs classified as unclassified *Cyclobacteraceae* or unclassified *Spirochaetaceae* were depleted at both mid-pH and low-pH sites. Also, 10 ASVs classified as *Endozoicomonas* were depleted in Poc-communities at either the mid-pH or the low-pH site. In Plob-communities, 53 and 36 ASVs were differentially abundant at mid-pH and low pH sites, respectively, with 22 ASVs being differentially abundant at both mid- and low-pH sites (see Table S2). Differentially abundant ASVs in Plob-communities were diverse, representing 26 bacterial families across 9 phyla. The ASV most enriched in Plob-communities at both mid-pH and low-pH sites was “*Candidatus* Amoebophilus,” and the ASV most depleted at both mid-pH and low-pH sites was *Nitrospira*. Seven ASVs, classified as *Endozoicomonas*, were significantly enriched at the low-pH site. Prus-communities had only four ASVs that were differentially abundant compared to the background-pH site: two ASVs, classified as *Endozoicomonas*, were significantly enriched at the low-pH site, and two ASVs, classified as *Algicola* and unclassified *Cellvibronaceae*, were significantly depleted at the low-pH site (see Table S2). No ASVs were significantly differentially abundant in all three coral species.

*Endozoicomonas* (order *Gammaproteobacteria*, family *Endozoicomonadaceae*) was the only bacterial taxon with differentially abundant ASVs present in all three coral species, but each coral species had different *Endozoicomonas* ASVs contributing to differences within that species (see Table S2). Therefore, the relative abundance of the genus *Endozoicomonas* (from all ASVs classified within this genus) was explored further. Poc-communities were dominated by *Endozoicomonas*, averaging 96.1 ± 0.8% of the total community at the background-pH site. *Endozoicomonas* abundance in Poc-communities decreased across the pH gradient, averaging 93.0 ± 1.7% and 67.4 ± 4.6% at the mid-pH and low-pH sites, respectively (Fig. 3b), but was significantly lower only at the low-pH site (Kruskal-Wallis; H = 20.54, *P* = 0.0001). Plob-communities at the low-pH site were distinct resulting from a significant increase in *Endozoicomonas* abundance, averaging 63.4 ± 3.6% of Plob-communities at the low-pH site, compared to 0.4 ± 0.1% and 0.01 ± 0.01% of Plob-communities at the background-pH and mid-pH sites, respectively (Kruskal-Wallis: H = 23.96, *P* = 0001; Fig. 3b). Prus-communities also had high abundance of *Endozoicomonas*, averaging 53.6 ± 4.5% at the background-pH site and 63.7 ± 4.8% at the mid-pH site, but Prus-communities also experienced a significant increase in *Endozoicomonas* abundance, averaging 87.2 ± 2.7%, at the low-pH site (Kruskal-Wallis: H = 17.37, *P* = 0.002; Fig. 3b).

Chloroplast-derived reads were a large proportion of the total reads from all samples (79.6%). In *P. eydouxi*, chloroplast-derived reads increased significantly across the pH gradient (Kruskal-Wallis: H = 9.92, *P* = 0.007) (see Fig. S3a). *Ostreobium* abundance in *P. eydouxi* samples also increased across the pH gradient, comprising 2.2 ± 1.0%, 96.2 ± 1.9%, and 89.8 ± 4.1% of the chloroplast-derived reads at the background- mid-, and low-pH sites (Kruskal-Wallis: H = 19.21, *P* = 0.0001) (see Fig. S3b). In *P. lobata*, chloroplast-derived read abundances were significantly less abundant at the mid-pH site (Kruskal-Wallis: H = 11.95, *P* = 0.002) (see Fig. S3c). *Ostreobium* comprised 68.0 ± 2.5%, 39.1 ± 2.2%, and 83.4 ± 3.1% of the chloroplast-derived reads at the background-pH, mid-pH, and low-pH sites, respectively, but there was a significant difference in *Ostreobium* abundance at the mid-pH site only (Kruskal-Wallis: H = 23.11, *P* = 0.0001) (see Fig. S3d). Chloroplast-derived reads in *P. rus* had similar abundance across sites (Kruskal-Wallis: H = 0.71, *P* = 0.703) (see Fig. S3e), and *Ostreobium* abundance, which was 91.4 ± 0.9% at the background-pH site, was also similar across the pH gradient (Kruskal-Wallis: H = 2.86, *P* = 0.239) (see Fig. S3f).

### Changes in calcification fluid pH across the pH gradient were coral species specific.

**FIG 4:**
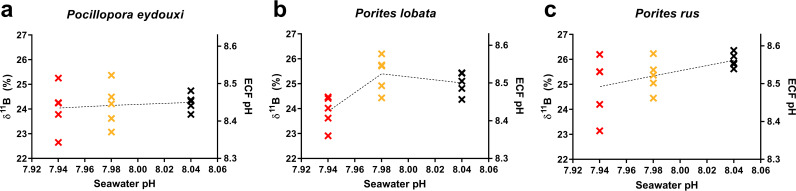
(a to c) Individual δ^11^B values and corresponding ECF pH estimates plotted against mean seawater pH values at background-pH (black), mid-pH (gold), and low-pH (red) sites for *Pocillopora eydouxi* (a), *Porites lobata* (b), and *Porites rus* (c) samples. Dotted lines intersect the mean values at each site.

Each coral species maintained its ECF pH higher than the external seawater pH at all sites, and *P. rus* maintained the highest ECF pH at each site (Fig. 4). In *P. eydouxi*, mean ECF pH decreased toward the vent (by 0.02 pH units), but there were no significant differences among sites (Kruskal-Wallis; H = 0.26, *P* = 0.878). In *P. lobata*, mean ECF pH increased modestly at the mid-pH site by 0.02 pH units and then dropped by 0.08 pH units at the low-pH site, compared to the background-pH site, but significant differences in mean ECF pH occurred only at the low-pH site (Kruskal-Wallis: H = 8.34, *P* = 0.015). In *P. rus*, mean ECF pH decreased by 0.08 pH units toward the vent, but like *P. eydouxi*, there were also no significant differences among sites chiefly as a result of increased variability in ECF pH values at the lower-pH sites (Kruskal-Wallis; H = 3.91, *P* = 0.142).

### Skeletal trace elements indicate exposure to vent emissions.

**FIG 5:**
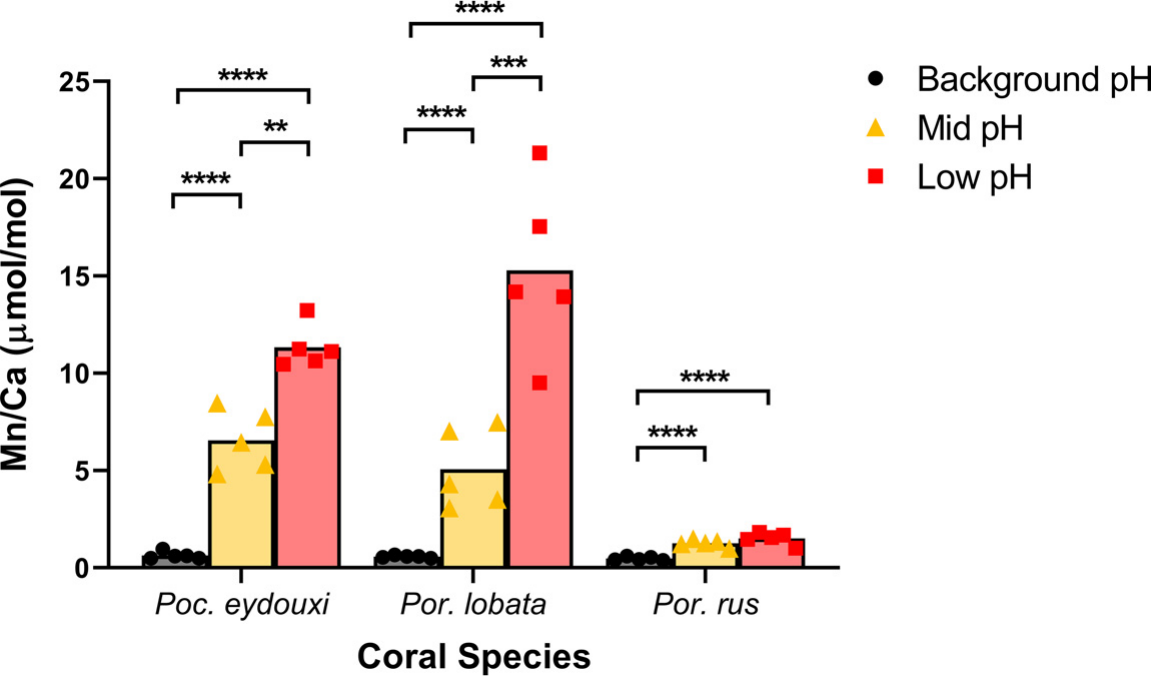
Mn/Ca ratio in coral skeletons. Data are presented as means with individual values. Black circles, background-pH site; gold triangles, mid-pH site; red squares, low-pH site. Sites within a species were compared using ANOVA with Tukey’s *post hoc* comparisons of log-transformed data. **, *P* < 0.01; ***, *P* < 0.001; ****, *P* < 0.0001.

Various trace elements present in the surrounding seawater are incorporated into the coral skeleton during calcification and can be used as proxies for local seawater conditions during calcification (36). Thus, the skeletal concentrations of trace metals that are emitted from the vent allow us to assess the exposure of individual colonies to vent emissions that are not spatially homogeneous within sites. Mn was the only trace element in coral skeletons that differed significantly across sites in all three coral species and serves as an indicator of the magnitude of vent exposure among and within sites (analysis of variance [ANOVA]: df = 2, *P* < 0.002) (see Table S3). At the background site, mean Mn/Ca was similar across all coral species (∼0.6 μmol/mol; Fig. 5). However, the mean Mn/Ca level increased in *P. eydouxi* and *P. lobata* by an order of magnitude at the mid-pH site (6.6 ± 0.7 μmol/mol and 5.1 ± 0.9 μmol/mol, respectively) and almost doubled in *P. eydouxi* and tripled in *P. lobata* again toward the low-pH site (11.3 ± 0.5 μmol/mol and 15.3 ± 2.0 μmol/mol, respectively). The mean Mn/Ca level in *P. rus* also increased significantly toward the vent, 1.3 ± 0.1 μmol/mol at the mid-pH site and 1.5 ± 0.1 μmol/mol at the low-pH site (Fig. 5). However, the Mn/Ca level in *P. rus* at both mid- and low-pH sites was significantly lower than that in the other two coral species at the mid-pH site (Fig. 5). In *P. lobata*, Fe/Ca also increased significantly toward the vent (ANOVA: df = 2; *P* = 0.0001), but no other trace element displayed significant differences across sites in either *P. eydouxi* or *P. rus* (see Table S3).

### Environmental drivers of bacterial community structure.

**FIG 6:**
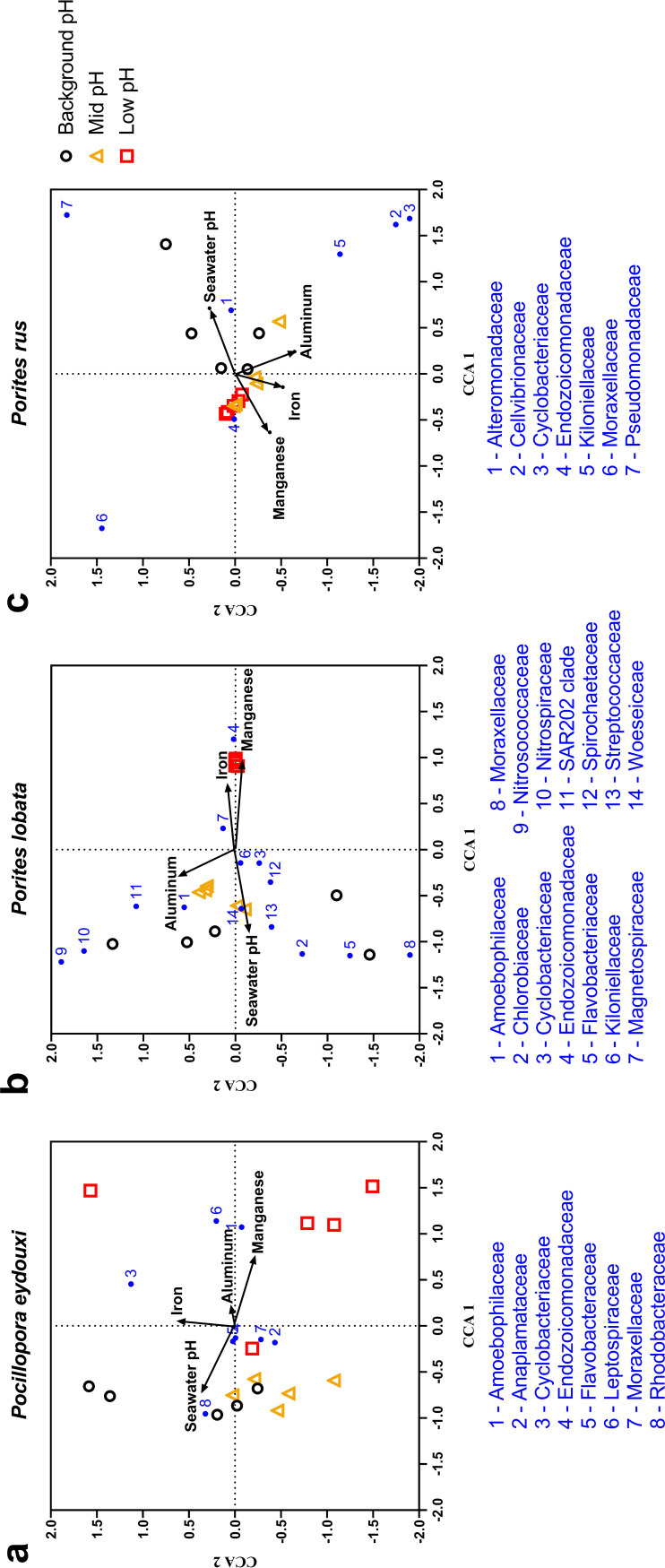
(a to c) Canonical correspondence analysis plots for *Pocillopora eydouxi* (a), *Porites lobata*, (b), and *Porites rus* (c). Ordination was obtained using vent-associated environmental variables (seawater pH and skeletal trace element concentrations) and the mean abundances of major bacterial families (>1% of total bacterial community within a coral species). All panels: black circle, background-pH site; gold triangle, mid-pH site; red square, low-pH site.

Particulate and dissolved Mn, Al, and Fe are known to emit from the Maug vent (37); therefore, CO_2_-driven reduction in seawater pH may not be the only environmental factor influencing coral-associated bacterial community structure at the mid- or low-pH sites. Canonical correspondence analysis (CCA) included 8 bacterial families for *P. eydouxi*, 14 bacterial families for *P. lobata*, and 7 bacterial families for *P. rus*. A Monte Carlo permutation test found that the CCA was robust in each coral species (*P* < 0.05), indicating a strong correspondence between relative abundance of major coral-associated bacterial families and predictor environmental variables (see Table S4). In all three coral species, CCA triplots showed high correspondence of seawater pH to background-pH-site samples, whereas Mn showed high correspondence to low-pH-site samples (Fig. 6). For *P. eydouxi*, seawater pH and Mn were the strongest predictor variables, with CCA axis 1 correspondence coefficients of −0.67 and +0.74, respectively (see Table S4). For *P. lobata*, seawater pH, Mn, and Fe were strong predictor variables, with CCA 1 correspondence coefficients of −0.89, +0.92, and +0.81, respectively (see Table S4). For *P. rus*, seawater pH and Mn were the strongest predictor variables, with CCA 1 correspondence coefficients of +0.71, and −0.63, respectively (see Table S4). No bacterial taxa clustered with a particular trace element in all the three coral species. In the *Porites* spp., most taxa have positive associations with increased seawater pH (Fig. 6).

## DISCUSSION

Microbiome restructuring in a new environment may lead to better stress tolerance in corals and/or may reflect host tolerance and maintenance of homeostasis under a new environmental regime (38–40), but microbiome restructuring has been shown to occur in a host-specific manner (41). Understanding host-specific microbial interactions under long-term OA is important to predict the future health and function of reefs, and we found that three coral species along a natural pH gradient exhibited changes in bacterial community structure and composition. In *P. eydouxi*, bacterial communities experienced increased community dispersion with lower seawater pH, following the Anna Karenina principle that dysbiotic individuals vary more in their microbial community composition than their healthy conspecifics (29). The potential breakdown of host-microbe interactions seen in *P. eydouxi* are consistent with other coral taxa, such as *Acropora millepora* and *Porites cylindrica*, at another naturally acidified reef in Papua New Guinea (26). However, *Pocillopora* spp. are proposed to be “microbial regulators” with relatively inflexible microbial associations, even under heat and nutrient stress (41, 42). The disruption to microbial communities seen in *P. eydouxi* at the low-pH site at Maug suggests that OA may represent a chronic environmental stress capable of budging even the most intransigent of coral-microbe associations. In contrast, in *Porites* spp., bacterial communities across the same pH gradient converged onto more tightly clustered communities with similar community composition, potentially reflecting processes of host acclimation and tolerance to OA conditions. Our data contrast with other studies, also from Papua New Guinea, that showed that bacterial communities in massive *Porites* spp. are resistant to changes in seawater pH (43). At Maug, *P. rus* displays a massive growth morphology but also displayed significant changes in bacterial community structure with lowered seawater pH. These differences in bacterial community flexibility of massive *Porites* spp. under OA conditions at Papua New Guinea and Maug may simply reflect species-specific responses to OA but could be useful for testing hypotheses regarding the role microbial flexibility in adapting to OA.

Conditions at Maug had a pronounced effect on the abundance of the bacterial genus, *Endozoicomonas*. This tissue-residing bacterium is found in various coral species across the globe (44) and is thought to be a symbiont (as opposed to a commensal) (45). We detect significant losses of *Endozoicomonas* toward the vent system in *P. eydouxi*. In contrast, both *Porites* species display significant increases in *Endozoicomonas* abundance as ambient seawater pH decreased. *P. lobata* in particular, increased the relative abundance of this taxon from 0% to almost 50% when in proximity to the vent. *Endozoicomonas* did not strongly correspond to other vent emissions (such as Mn, Fe, or Al), suggesting that lowered seawater pH, or the coral host’s response to lowered seawater pH, has a stronger influence on its abundance. Interestingly, different *Endozoicomonas* ASVs were enriched or depleted in each coral species, suggesting species-specific associations between different *Endozoicomonas* spp. and their coral hosts. Loss of *Endozoicomonas* in response to OA has been described in other coral species, such as *A. millepora* and even in massive *Porites* spp. (26, 43, 46), but our study documents gains of *Endozoicomonas* under low-pH conditions. Even though *P. eydouxi* experienced losses in *Endozoicomonas*, this genus was still the dominant bacterial taxa at the low-pH site. Gains in *Endozoicomonas* in both *Porites* species could suggest that *Endozoicomonas* may be beneficial to these coral under OA conditions. Alternatively, low-pH conditions may be lowering the *Porites* corals ability to control the growth of these intercellular bacteria. Either way, it raises the question as to why some coral species lose abundance of this bacterial taxon with OA and highlights the potential of this bacterial genus as an indicator of tolerance to OA.

OA is likely directly and indirectly influencing the structure and composition of coral-associated bacterial communities. The changes seen in *Endozoicomonas* abundance, for example, are likely due to indirect effects because *Endozoicomonas*, as a tissue-residing bacterium, would not be exposed to seawater. Rather, changes to host physiology in response to OA likely are influencing *Endozoicomonas* abundance. In contrast, mucus-associated and skeleton-associated bacteria do interact with seawater; thus, the direct impacts of changing seawater pH would be most evident in these communities. Because whole fragments were processed for amplicon sequencing, we cannot tease apart these potential direct versus indirect effects or differentiate patterns that may be driven by bacterial localization. Future studies should consider these partitions in coral-associated microbial communities to reveal more nuanced insights into the impact of OA on coral microbiome.

OA is also known to alter coral interactions with skeleton-associated eukaryotic endophytes. In particular, OA leads to higher abundance of the green algae *Ostreobium* in *Porites* skeletons, leading some to describe it as a harmful bioeroder (47, 48). However, *Ostreobium* is commonly found in the skeletons of living corals and provides photoassimilates to coral during thermal bleaching, leading others to suggest it is a beneficial coral symbiont (49). Assessment of eukaryotic microalgae communities in corals is best addressed by using 23S or ITS2, rather than 16S, rRNA sequencing, but we were able to classify *Ostreobium* sequences with high confidence using the Protist Ribosomal Reference database (50). Similar to previous studies, *P. eydouxi* and *P. lobata* at Maug displayed increased abundances of *Ostreobium* with decreasing seawater pH, and *P. eydouxi* had similar (but high) abundances of *Ostreobium* across the pH gradient. The high *Ostreobium* abundance, especially at the low-pH site, in all corals suggests that it may be acting as a harmful bioeroder.

We also document genus-specific responses in calcification physiology in response to long-term low-pH conditions. In *P. eydouxi*, calcification physiology was highly regulated across all seawater conditions, whereas both *Porites* experienced a mean decrease in ECF pH near the vent. *P. rus* has been suggested as an OA-resistant coral (13, 14), and this species appears the most OA tolerant in this study in terms of calcification physiology, maintaining the highest ECF pH of all three species across all sites and experiencing only a slight decrease in ECF pH across the pH gradient. The variable response in pH upregulation in *P. rus* (and other coral species) may reflect localized differences in exposure to vent emissions, as discussed below, or a variable response to low-pH conditions by different individuals. Our data show that different coral species have varying capability to raise ECF pH and maintain calcification rates under OA. Expanding this analysis to other coral species and understanding the genetic/molecular mechanisms will help identify OA resilient corals species and/or populations.

Species-specific upregulation of ECF pH in response to OA has been documented but during short-term experiments (19, 51). Similarly, species-specific changes in coral bacterial communities in response to OA conditions have been documented but under far more extreme pH reductions (pH 7.5 or less) (26, 33, 52). Here, we document changes in ECF pH and the composition of coral bacterial communities with long-term *in situ* exposure to conditions (−0.1 pH units) projected to occur at the end of this century under midrange emissions scenarios (10). Seawater pH on coastal coral reefs may decline faster than open ocean predictions, especially for coral reefs in lagoons or enclosed bay with less mixing, because community metabolism and local watershed influences can drive large declines and/or high variability in local seawater pH (53, 54). Therefore, coral reefs around the globe may soon experience, or may already experience, the levels of OA stress needed to impact calcification and microbial symbiosis.

The different responses to OA stress described at Maug may have important long-term consequences to coral population dynamics. ECF pH upregulation may allow *P. eydouxi* to sustain normal calcification in spite of further OA, but bacterial community destabilization below a pH threshold may increase susceptibility to other environmental disturbances or cause mortality by allowing opportunistic pathogens to proliferate or reduce energy supply. In contrast, *P. lobata* and *P. rus* may experience similar reductions in calcification or skeletal density as other corals under OA conditions but may benefit from a more stable bacterial community. Given increasing seawater temperatures and recurrent global bleaching events, a stable, mutualistic bacterial community will be an important factor in reef persistence even at relatively pristine reefs like those found at Maug.

Investment trade-offs can occur among different physiological functions in corals under stress, including OA (55). For example, spawning female colonies of *Astrangia pocluata*, which require more energy for the production of gametes, experienced decreased calcification under OA conditions compared to spawning male colonies (56). However, it is unclear whether other physiological functions, such as regulation of bacterial communities, also impact calcification sensitivity to ocean acidification, or vice versa. Short-term OA experiments reveal complex coral calcification and bacterial symbiosis responses. Experimental OA reduced both calcification rates and microbial nitrogen fixation rates in *Seriatopora hystrix* (34), and dual OA and temperature stress destabilized the bacterial community and decreased calcification in a thermally sensitive coral (*Acropora millepora*) (31), suggesting that both calcification and bacterial symbiosis are impacted negatively with OA. In a more thermally tolerant coral (*Turbinaria reniformis*), dual OA and temperature stress neither reduced calcification nor destabilized bacterial communities, suggesting that both physiological processes can be maintained in some corals during short-term stress (31).

Given the physical separation of mucus- and tissue-associated bacteria from the host tissues undergoing calcification, a direct link between bacterial community structure/composition and calcification physiology is unlikely. However, the dichotomy in ECF pH upregulation and bacterial community structure seen in this study reveals a potential investment trade-off by the coral host under long-term OA that warrants further investigation. Corals have cellular mechanisms to optimize calcification conditions in the ECF, which consume ATP (17, 57, 58), and the energy requirements to elevate ECF pH relative to seawater pH increase exponentially under increasing OA (59). It remains poorly understood how corals select for and regulate their bacterial communities, but it is thought to involve the composition and shedding of mucus (60–62) and/or interactions with immune defenses (63–65), both energetically costly functions (66–69). Thus, by maintaining high ECF pH at the Low pH site, *P. eydouxi* is likely investing more energy into calcification, compared to *P. lobata and P. rus*, potentially at the cost of resources needed to maintain stable bacterial communities. We did not measure skeletal density or linear extension rates in the corals collected for this study; however, Enochs et al. report depressed calcification and linear extensions rates for *Porites* spp. at Maug (70). Controlled, manipulative experiments will be needed to mechanistically link changes in coral bacterial communities and calcification physiology under long-term OA conditions to determine how different coral species may invest in OA tolerance.

Mn/Ca in our coral skeletons provides a useful covariate to approximate long-term vent exposure in lieu of discrete seawater pH measurements taken at the time and place each coral colony was sampled within the sites. *P. eydouxi* and *P. lobata* display strong increases in skeletal Mn/Ca across sites, suggesting a robust gradient of vent exposure across the three collection sites. *P. rus* displayed a much weaker signal. Interspecific differences in Mn incorporation could explain lower Mn/Ca values in *P. rus* at mid- and low-pH sites (71, 72). However, the natural distribution of corals within each site and heterogeneous distribution of vent emissions across the reef could also explain the weaker Mn signal in *P. rus*. At the low-pH site, *P. rus* colonies form a large, monospecific wall with high relief that grows to the edge of the vent zone. In contrast, *P. eydouxi* and *P. lobata* colonies are sparsely distributed throughout the vent zone (R. Day, unpublished data). Thus, the *P. rus* colonies may not be experiencing as great an OA gradient compared to *P. eydouxi* and *P. lobata*.

Previous studies have stressed the need to account for other hydrothermal emissions (such as metals) as potential confounding variables when utilizing volcanic vents as natural laboratories of future OA conditions (73, 74). Sulfur-rich compounds have not been detected in proximity to the vent, but Maug caldera does emit dissolved and particulate Mn, Al, and Fe (37) that, in addition to increased pCO_2_, may affect host physiology and bacterial community structure. To address this concern, we utilized CCA on a subsample of our bacterial community data and found that seawater pH was the strongest predictor variable to bacterial community structure in all three coral species, followed by Mn and Fe. Both Mn and Fe may impact bacterial community structure because these trace elements are used in various biological processes, including photosynthesis, redox reactions, nutrient acquisition, cell adhesion and biofilm formation, and these metals can be toxic to microbes (75, 76). Marine bacterial taxa that are known to be Mn or Fe reducers or oxidizers (77–79) or taxa known to associate with Mn-enriched marine environments (80, 81) were not abundant (<1% of total bacterial reads) in any coral sample at any site. For example, *Chlorobiaceae* require Fe for anoxygenic photosynthesis, but this abundant family was not positively correlated with Fe concentrations. These data suggest that trace element emissions from the Maug vent do not directly alter coral-associated bacterial communities. However, the direct impacts of these other vent emissions on mucus-associated bacteria may be difficult to detect. In addition, we cannot currently assess whether other vent emissions could be affecting bacterial community structure and/or composition via impacts on host physiology.

Utilizing both 16S rRNA gene amplicon sequencing and biogeochemistry analysis provides deeper insight into the long-term impacts of OA on coral physiology. The species-specific responses to long-term OA described here and their potential ecological implications highlight the need to understand the mechanisms behind differential susceptibility and resilience of reef-building corals to OA. This study did not investigate the response of an important member of the coral holobiont (i.e., *Symbiodiniaceae*) or seek to answer how the responses of the coral holobiont influence each other. With the ability to control for volcanic influence using skeletal biogeochemistry approaches, it is imperative to leverage the high research potential of CO_2_ vents, such as Maug caldera, to better understand how changes in coral holobiont physiology will impact health and function of reef ecosystems as global CO_2_ emissions continue to increase.

## MATERIALS AND METHODS

### Study site and sample collection.

Maug is an uninhabited group of three islands located in the Northern Mariana Islands (20°1N, 145°13E) (Fig. 1a) that make up a sunken volcanic caldera. The reefs studied within the caldera are shallow (∼9-m depth), with localized submarine volcanic vents that bubble CO_2_ creating a localized gradient of reduced pH and aragonite saturation state (37, 47). In addition to CO_2_, the volcanic vent at Maug emits particulate and dissolved iron, manganese, and silica (37). No significant concentrations of sulfur-rich compounds such as sulfate, sulfide, or hydrogen sulfide have been detected in proximity to the vent (37). Three study sites were previously established along this 1,500-m gradient and are labeled as background pH, mid pH, and low pH (Fig. 1b). Previous work conducted by Enochs et al. (70) characterized the mean seawater pH, mean pCO_2_, and benthic composition at these three discrete sites in order to capture the temporal variability in carbonate chemistry, described briefly below. The mean seawater pH levels (± the standard deviations) measured over a 3-month interval (*n* = 3,984 measurements) at these sites were 8.04 ± 0.016, 7.98 ± 0.027, and 7.94 ± 0.051, respectively, with the low-pH site reaching a minimum of pH of 7.72. The mean pCO_2_ levels (± the standard deviations) measured from discrete water samples over a 2-day period were 401.3 ± 4.61, 441.2 ± 21.23, and 502.0 ± 29.67 μatm, at the background-pH, mid-pH, and low-pH sites, respectively (70). Percent coral cover also decreases from >50 to ∼20% to <1% at the background-pH, mid-pH, and low-pH sites, respectively (70). Temperature, measured over the same 3-month period, did not vary significantly across sites (70). Light, measured as daily dose of photosynthetically active radiation, was reported to decrease significantly across the pH gradient: 10.9 ± 2.46, 9.5 ± 2.46, and 6.5 ± 4.16 mol photons per m^2^ at the background-pH, mid-pH, and low-pH sites, respectively (70). However, light levels were not found to be a major driver of benthic community structure at Maug (70), and the high variability in light levels, especially at the low-pH site, suggest that more measurements over a longer period of time (light measurements data were collected over the course of just 2 days) may be necessary to better understand differences in light regime across sites.

One fragment was collected from 10 colonies of three coral species (*Pocillopora eydouxi*, *Porites lobata*, and *Porites rus*) via hammer and chisel at each of the three sites via SCUBA in 2014. At Maug, *P. eydouxi* forms discrete branching colonies, *P. lobata* forms discrete mounding colonies, and *P. rus* forms massive or discrete mounding colonies. Fragments were collected from an accessible terminal branch or lobe at the top of each colony. At each site, coral fragments were collected from colonies close to the location of seawater chemistry measurements, within an area of ∼50 m^2^. At the low-pH site, the massive colonies of *P. rus* are on the immediate south perimeter of the vent compared to the samples collected from *P. eydouxi* and *P. lobata*, which were distributed throughout the vent. Coral fragments were placed in individual bags and snap-frozen in liquid nitrogen at the surface. Frozen coral fragments were kept in liquid nitrogen at the National Institute of Standards and Technology’s Marine Environmental Specimen Bank until further processing. Corals were collected under NOAA Pacific Islands Fisheries Science Center permits, approved by the Commonwealth of the Northern Mariana Islands Department of Fish and Wildlife, and the collection was conducted in accordance with applicable rules and regulations governing fieldwork and sample collection at the study site.

### Skeletal biogeochemistry analysis.

Preprocessing for skeletal biogeochemistry included subsampling half of the coral samples (*n* = 5 from each coral species at each site) under liquid nitrogen, followed by lyophilization for 24 h. Dry coral samples were then scraped using a scalpel to collect the exterior layer of skeleton most closely associated with living tissue. An aliquot of the coral powder (∼100 mg) was treated twice with a 5% sodium hypochlorite solution at room temperature for 24-h periods to remove the soft tissue. Skeletal samples were then washed with boron-free Milli-Q water (>18.2 MΩ·cm) and then further lyophilized to remove moisture. Carbonate powders were further subsampled (∼5 mg) and subjected to additional oxidative cleaning in warm 1% H_2_O_2_ (buffered in ammonium hydroxide) to chemically remove remaining organic matter. These samples were then given a weak acid leach (0.0005 M HNO_3_) to remove any readsorbed ions. Once cleaned, samples were dissolved in a minimal volume of Optima 0.5 M HNO_3_ (Fisher Scientific).

Trace elements in each dried coral skeleton were measured using Thermo Element II inductively coupled plasma mass spectrometry (ICP-MS) at the National Institute of Standards and Technology (NIST). Dissolved samples were diluted in 0.5 M HNO_3_ to 80 μg/g [Ca] for analysis. Samples were run using multimode detection and low and medium mass resolution using a method modified from Marchitto (82). Multielement external calibration using gravimetrically prepared matrix-matched standards were used to quantify Li, B, Al, Na, Mg, V, Mn, Fe, Co, Ni, Cu, Zn, Rb, Sr, Mo, Cd Sb, Ba, Nd, Pb, and U analytes relative to Ca. Blanks were run between each sample/standard, which were used to blank-correct counts of each element before ratios were determined. The limit of detection for each element for each run was determined as three standard deviations above the mean of the blanks. The percent relative standard deviation was calculated for each elemental ratio from replicate measurements (*n* = 37) of a matrix-matched control material (NIST RM 8301 Coral) to assess analytical precision (83). The majority of the measured trace elements had an uncertainty of ≤±2% (Li/Ca, Al/Ca, Na/Ca, Mg/Ca, Co/Ca, Cu/Ca, Rb/Ca, Sr/Ca, Cd/Ca, Sb/Ca, Ba/Ca, Nd/Ca, Pb/Ca, and U/Ca), with B/Ca, Fe/Ca, Ni/Ca, and Zn/Ca ≤ ±5% and V/Ca, Mn/Ca, and Mo/Ca ≤ ±10%.

Boron isotopes analysis was conducted according to established methods (84, 85). Boron in the remaining solutions (∼200 ng of B) was separated from the carbonate matrix using 20-μl microcolumns (Amberlite IRA 743 boron-specific anionic exchange resin). After elution of the boron fraction, additional elutions were checked to ensure > 99% of sample boron was recovered in the sample. The purified boron samples were diluted to 100 ppb [B] for analysis. The δ^11^B of samples were measured on a multicollector Nu Plasma II MC-ICP-MS against NIST SRM 951a. The accuracy and precision of δ^11^B results were assessed using carbonate standards NIST RM 8301 Coral and JCp-1. Measured values for these reference materials during sample analysis were, respectively, 24.35‰ ± 0.26‰ (2 standard deviations; *n* = 34) and 24.06‰ (*n* = 2), which were within uncertainty of interlaboratory consensus values (83, 86). Seven total procedural blank measurements were made alongside samples in this study (average of 104 pg of boron). These blanks were found to be small (<0.06% of sample boron) resulting in minimal impact on δ^11^B sample results (i.e., less than analytical uncertainty), hence a total procedural blank correction was not applied.

### Extracellular calcifying fluid pH calculations.

The ECF pH of each sample was estimated from measured skeletal δ^11^B using the following equation (87, 88):

$$\text{pH = p}K_{\text{B}}^{*}-\text{log}\left(-\frac{\delta^{11}{\text{B}}_{\text{sw}}\;-\;\delta^{11}{\text{B}}_{\text{coral}}}{\delta^{11}{\text{B}}_{\text{sw}}\;-\;\alpha_{\text{B}}\delta^{11}{\text{B}}_{\text{coral}}\;-\;1,000(\alpha_{\text{B}}\;-\;1)}\right)$$

where α_B_ (1.027) is the fractionation factor between boric acid and borate (89), δ^11^B_sw_ (39.61) is the boron isotopic composition of seawater (90) and p*K*_B_* (8.54) is the dissociation constant of the two boron species calculated using the Seacarb package in R with site-specific temperature of 30°C and salinity of 35 ppt (70). Analytical uncertainty on δ^11^B measurements contributes to a <0.02 pH unit shift in calculated internal pH.

### Bacterial community analysis.

Preprocessing included subsampling from each coral fragment (*n* = 90) and subsequent homogenization (minimum of 2 min at 25 reps per second) all under liquid nitrogen using a Retsch Cryomill (Retsch GmbH, Haan, Germany). An aliquot of each coral homogenate (∼300 mg) was preserved in 1 ml of TRIzol reagent and stored at −80°C until nucleic acid extraction. Preserved DNA was separated from RNA using organic solvent phase separation with chloroform, and subsequent DNA extraction was conducted using back-extraction buffer (BEB; 4 M guanidine thiocyanate; 50 mM sodium citrate; 1 M Tris [pH 8.0]) (91). After removal of the aqueous phase containing RNA, BEB was added to the organic phase and interphase and then incubated at room temperature for 10 min. Samples were then centrifuged at 13,200 rpm for 15 min at 4°C. The upper, aqueous phase (now containing DNA) was removed, an equal volume of 100% isopropanol was added, and the samples were incubated overnight at −20°C. The samples were then centrifuged at 13,200 rpm for 30 min at 4°C to pellet DNA. The supernatant was removed, and the DNA pellets were washed twice with 70% ethanol and then resuspended in Nanopure water. Extracted DNA was quantified using a NanoDrop spectrophotometer and submitted to the BioAnalytical Services Laboratory at the Institute of Marine and Environmental Technology for high-throughput sequencing of V1-V3 hypervariable regions of the 16S rRNA gene using 27F (5′-AGAGTTTGATCCTGGCTCAG-3′) and 534R (5′-ATTACCGCGGCTGCTGG-3′) on an Illumina Mi-Seq (paired-end 2 × 300 read) platform.

Sequence analysis was conducted using QIIME2 v. 2019.10 pipeline (92). Paired-end, demultiplexed reads were quality filtered, trimmed of poor-quality bases, dereplicated, chimera filtered, pair merged, and identified as amplicon sequence variants (ASVs) using the DADA2 plug-in (93). Taxonomy was assigned by training a naive-Bayes classifier on the V1-V3 region of the 16S rRNA gene in the SILVA version 138 database (94) using the feature-classifier plugin (95) to match the primers used. Nonprokaryotic ASVs (i.e., mitochondria, eukaryote, and unassigned) were then removed. Sequences classified as “chloroplast” were also removed from the analysis of bacterial sequences but were saved as a separate data set. Sequences identified as chloroplast via the SILVA database were further classified using the Protist Ribosomal Reference (PR^2^) database (50). Rarefied bacterial ASV tables (rarefied to 1,100 reads per sample) were used to calculate alpha-diversity metrics and to conduct beta-diversity analyses using weighted UniFrac distance matrices. For each coral species, alpha rarefaction curves of bacterial ASVs did reach a plateau, indicating sufficient sampling depth at to 1,100 bacterial reads per sample (see Fig. S3).

### Statistics.

Skeletal trace element ratios were log transformed to meet assumptions of normality and then compared among sites, separated by species, using one-way ANOVA with Tukey’s *post hoc* comparisons and Bonferroni correction. Data for Mn were further compared among site and species groups as a proxy of vent exposure, using one-way ANOVA with Tukey’s *post hoc* comparisons. Due to unequal variances in other data sets, even after transformation, a nonparametric Kruskal-Wallis (with Dunn’s *post hoc* comparisons and Bonferroni correction) was used to compare ECF pH, alpha-diversity metrics, beta-diversity metrics, and relative abundances of major microbial taxa among sites (within each coral species). All data are represented as means ± the standard errors, unless stated otherwise.

The weighted UniFrac distance matrix was used to calculate the beta diversity to assess dispersion in bacterial communities between samples at each site (within each species). The weighted UniFrac distance matrix was also used to construct principal-component analysis (PCoA) plots to visualize differences in bacterial community structure between sites. PCoA was conducted for all samples and then for each species individually. Pairwise analysis of similarities (ANOSIM) was used to test for significant differences in bacterial communities among sites. To assess differences in ASV abundance across the pH gradient, the R package DESeq2 (v1.26.0) was used to fit a negative binomial model using the unrarefied ASV table for each species, and then Wald tests were used to test for differences in taxon abundance between mid- or low-pH sites versus the “control” (background-pH site). Benjamini-Hochberg false discovery rate tests were used to account for multiple comparisons, and ASVs with *P* values of <0.05 were identified as significantly differentially abundant.

To test which environmental variables (vent-associated trace elements and external seawater pH) have significant relationships to bacterial community structure, canonical correspondence analysis (CCA) was performed using the program PAST v3 (96). Inputs for CCA analysis include concentrations of trace elements with significant differences among sites or known to be emitted from the vent, the mean seawater pH of each site as measured previously (47), and the relative abundances of major bacterial families (>1% of total bacterial community). A Monte Carlo test with 999 permutations was carried out to ensure the significance of the canonical axes.

### Data availability.

The raw sequence data files have been submitted to the NCBI Sequence Read Archive under accession number SRP174887. Data files, including concentrations of trace elements, an ASV table, an ASV taxonomy assignment, and R script for DESeq analysis, are available on Figshare at https://figshare.com/account/home#/projects/88106.

## Supplementary Material

Supplemental file 1
