# Supplementary material for: Dichotomy between Regulation of Coral Bacterial Communities and Calcification Physiology under Ocean Acidification Conditions
Source: Appl Environ Microbiol. 2021 Feb 26;87(6):e02189-20. doi: 10.1128/AEM.02189-20 (PMC8105028; doi:10.1128/AEM.02189-20)
Supplement: Supplemental file 1 [file AEM.02189-20-s0001.pdf]

Dichotomy between regulation of coral bacterial communities and calcification physiology under ocean acidification conditions

A. Shore[1,2], R. D. Day[3,4], J. A. Stewart[4,5], C.A. Burge[1*]

[1]Institute of Marine and Environmental Technology, University of Maryland Baltimore County, Baltimore, MD, USA
[2]Department of Biology, Farmingdale State College, Farmingdale, NY, USA
[3]Marine Science and Nautical Training Academy, Charleston, SC
[4]Hollings Marine Laboratory, National Institute of Standards and Technology, Charleston, SC, USA
[5]School of Earth Sci. Univ. of Bristol, Queens Road, Bristol, BS8 1RJ, UK
*Corresponding author: colleenb@umbc.edu

Supplemental Materials

Supplemental Table S1. ANOSIM comparisons of the weighted UniFRAC distance matrix values for bacterial communities by coral species

Supplemental Table S2. Differentially abundant bacterial ASVs in coral determined by DESeq2.

Supplemental Table S3. Statistical comparisons of log-transformed trace element concentrations in coral skeleton across sites.

Supplemental Table S4. Results of Canonical Correspondence Analysis of each coral species in relation to vent associated predictor variables.

Supplemental Figure S1. Principle Coordinate analysis (PCoA) of the weighted UniFrac distance matrices for all three coral species.

Supplemental Figure S2. Mean percent abundance of chloroplast-derived versus bacterial sequences for a) *Pocillopora eydouxi*, c) *Porites lobata*, and e) *Porites rus* coral samples at Background pH, Mid pH and Low pH sites.

Supplemental Figure S3. Alpha rarefaction curve of observed ASVs by sequencing depth for all three coral species.

Supplemental Table S1. ANOSIM comparisons (999 permutations) of the weighted UniFRAC distance matrix values for all sampled coral-associated bacterial communities based on corals species. Significant differences are shown in **bold**.

Comparison	Global R	P value
Pocillopora eydouxi* vs. *Porites lobata	**0.407**	**0.001**
Pocillopora eydouxi* vs. *Porites rus	**0.830**	**0.001**
Porites lobata* vs. *Porites rus	**0.297**	**0.005**

Supplemental Table S2. Bacterial Amplicon Sequence Variants (ASVs) within coral samples differing significantly ($p < 0.05$) in abundance between Sites as determined by DESeq2. A positive Log2 Fold Change indicates higher abundance in the first comparison.

Comparison	base Mean	Log2 Fold Change	lfcSE	Stat	Adjusted p-value	ASV ID	Genus
*P. eydoux*i: Low pH vs. Background pH	62.0310	24.1022	3.5341	6.8199	7.11E-11	f990baa42ffa60592c8ff12d93ba2e5b	Candidatus *Amoebophilus*
*P. eydoux*i: Low pH vs. Background pH	3.8488	-22.1889	3.5365	-6.2743	2.28E-09	331e144270b4f1fb0759d2f89da878a4	unclassified Spirochaetaceae
*P. eydoux*i: Low pH vs. Background pH	14.7833	-23.9933	3.5345	-6.7883	8.04E-11	7bde5aa2d8f23e0dd7f5208d075eb254	unclassified Cyclobacteriaceae
*P. eydoux*i: Low pH vs. Background pH	17.1242	-24.1300	3.5344	-6.8272	7.11E-11	2b9a7aabc6deb4e894ffc37bfc6f6a36	unclassified Cyclobacteriaceae
*P. eydoux*i: Low pH vs. Background pH	17.3897	-24.1834	3.5344	-6.8423	7.11E-11	70c7fb1f618a11185e374ec412d8912f	unclassified Spirochaetaceae
*P. eydoux*i: Low pH vs. Background pH	22.4740	-24.5750	3.5343	-6.9534	3.97E-11	b67a7c4d3284e20b836491d8f330b40d	unclassified Cyclobacteriaceae
*P. eydoux*i: Low pH vs. Background pH	68.7931	-25.2142	3.5341	-7.1346	1.26E-11	b25f83e7c5b43ce2f83c50359d119634	*Endozoicomonas*
*P. eydoux*i: Low pH vs. Background pH	69.5892	-25.6542	3.5340	-7.2593	6.07E-12	a84e6bd6dbb88bdc4663c85a5dbad410	*Endozoicomonas*
*P. eydoux*i: Low pH vs. Background pH	154.6989	-26.2099	2.6353	-9.9456	1.03E-21	b5b74c2b4e2c91f1196dd342119f9fa7	*Endozoicomonas*
*P. eydoux*i: Low pH vs. Background pH	145.9548	-26.6274	3.2608	-8.1660	6.22E-15	ee444a422716261964850ec423ffa912	*Endozoicomonas*
*P. eydoux*i: Low pH vs. Background pH	175.8576	-26.7574	3.0488	-8.7764	4.39E-17	215208b3e07f66b1b92f61bd1f7b97bf	*Endozoicomonas*
*P. eydoux*i: Low pH vs. Background pH	424.6626	-27.7064	2.5396	-10.9098	8.07E-26	449ad69b1b37669430b2517cdec12f46	*Endozoicomonas*
P. eydouxi: Mid pH vs. Background pH	62.0310	22.5559	3.6282	6.2167	6.73E-09	f990baa42ffa60592c8ff12d93ba2e5b	Candidatus *Amoebophilus*
P. eydouxi: Mid pH vs. Background pH	3.8488	-21.5451	3.6364	-5.9248	3.68E-08	331e144270b4f1fb0759d2f89da878a4	unclassified Spirochaetaceae
P. eydouxi: Mid pH vs. Background pH	8.1091	-22.5953	3.6289	-6.2265	6.73E-09	268c8b02632a1e621feb4ce79b615935	*Endozoicomonas*
P. eydouxi: Mid pH vs. Background pH	14.7833	-23.3245	3.6345	-6.4175	2.45E-09	7bde5aa2d8f23e0dd7f5208d075eb254	unclassified Cyclobacteriaceae
P. eydouxi: Mid pH vs. Background pH	17.3897	-23.5339	3.6344	-6.4753	2.01E-09	70c7fb1f618a11185e374ec412d8912f	unclassified Spirochaetaceae
P. eydouxi: Mid pH vs. Background pH	17.1242	-23.5633	3.6344	-6.4834	2.01E-09	2b9a7aabc6deb4e894ffc37bfc6f6a36	unclassified Cyclobacteriaceae
P. eydouxi: Mid pH vs. Background pH	22.4740	-23.9003	3.6343	-6.5764	1.70E-09	b67a7c4d3284e20b836491d8f330b40d	unclassified Cyclobacteriaceae
P. eydouxi: Mid pH vs. Background pH	132.1391	-26.0545	3.6339	-7.1698	3.98E-11	e53ca5c125c31807f20c648445efab19	*Endozoicomonas*
P. eydouxi: Mid pH vs. Background pH	160.3477	-26.2672	3.6339	-7.2284	3.98E-11	3a51d417b62db6f3b3968d1aac0401a0	*Endozoicomonas*
P. lobata: Low pH vs. Background pH	33.5972	28.0425	3.5653	7.8655	1.22E-12	edbf61ceeed7720011cfb57d86d29726	Candidatus *Amoebophilus*
P. lobata: Low pH vs. Background pH	51.2149	25.6027	3.6340	7.0454	1.16E-10	3a4ab8f7eaba74e331c58bace1b6a052	*Endozoicomonas*

P. lobata: Low pH vs. Background pH	33.1177	24.7075	3.6340	6.7989	3.88E-10	ff15c7503cf6a803f60e755d75a3ae74	*Tistlia*
P. lobata: Low pH vs. Background pH	53.7898	24.5729	3.6340	6.7620	4.50E-10	7b6144c385ab6190a60384fa2a07105a	*Endozoicomonas*
P. lobata: Low pH vs. Background pH	41.2450	24.3885	3.6340	6.7112	5.81E-10	0e6ffcff41df7533661b4658999d5e97	*Endozoicomonas*
P. lobata: Low pH vs. Background pH	21.2955	24.0101	3.6342	6.6067	8.34E-10	3dfe08a40c12c92450733833a882eb94	*Tistlia*
P. lobata: Low pH vs. Background pH	14.3717	23.2219	3.6343	6.3896	2.62E-09	fdee3f3f9d1c29d4e56a2d915877176d	*Endozoicomonas*
P. lobata: Low pH vs. Background pH	51.1636	22.4213	3.3727	6.6478	7.22E-10	61124b9645d64cfef82a94e2b9bcf1c0	unclassified Cyclobacteriaceae
P. lobata: Low pH vs. Background pH	72.9649	22.0154	3.6345	6.0574	2.08E-08	95edabdf27b0c79327ace1b9ecce6609	*Tistlia*
P. lobata: Low pH vs. Background pH	14.8238	20.9478	3.6353	5.7623	1.06E-07	6e0d33c30f0a0700fb9d4488aa2fb1f4	Candidatus *Amoebophilus*
P. lobata: Low pH vs. Background pH	24.8058	19.9993	3.6367	5.4993	4.35E-07	98451532b5cdf21fb94465d7b6f4fa35	*Woeseia*
P. lobata: Low pH vs. Background pH	137.0218	11.3176	2.9321	3.8599	0.0010727	7636f129a0717af89fd0f51bc8896832	*Endozoicomonas*
P. lobata: Low pH vs. Background pH	133.8766	11.2841	2.9301	3.8511	0.001081	2862bf4d03d0ae0d20dc82d1510cf909	*Endozoicomonas*
P. lobata: Low pH vs. Background pH	77.0126	10.4864	3.1999	3.2771	0.0089003	213e09372189fc41e6455d0cf7b8e2f2	*Endozoicomonas*
P. lobata: Low pH vs. Background pH	22.3781	-10.8179	3.6906	-2.9312	0.0279426	930218db41fbce6afaddf903e1ae58fe	*Woeseia*
P. lobata: Low pH vs. Background pH	18.9166	-13.5203	3.6906	-3.6634	0.0022265	7dcd0d9e009fa95048f75bb1b12b9812	PS1 clade
P. lobata: Low pH vs. Background pH	46.9970	-14.6049	3.6906	-3.9573	0.000738	c2433508e8802e7ea43ef214fc813f62	*Tistlia*
P. lobata: Low pH vs. Background pH	30.4773	-15.2006	3.6906	-4.1187	0.0003822	ed914d7621c19d2468decd160bc36873	unclassified Kiloniellaceae
P. lobata: Low pH vs. Background pH	33.0470	-15.9526	3.6906	-4.3225	0.0001596	229657d69e3fe30bc94a46697e5616cd	SAR324 clade
P. lobata: Low pH vs. Background pH	134.2904	-17.0447	3.6906	-4.6184	4.13E-05	b059a6d42b6c88bab91bdd9413c8bc34	Candidatus *Amoebophilus*
P. lobata: Low pH vs. Background pH	21.5247	-19.3172	3.6012	-5.3642	8.97E-07	4c6f175048165bfbbe4e87fa85f77fc3	unclassified Kiloniellaceae
P. lobata: Low pH vs. Background pH	21.1736	-20.1792	3.6419	-5.5408	3.69E-07	cca9b789aee2f107478fd49cfaa62e88	unclassified Blattabacteriaceae
P. lobata: Low pH vs. Background pH	101.2087	-21.7413	3.6299	-5.9895	3.03E-08	c297ccd20cdda6ed4d64c77b4e894c27	*Prosthecochloris*
P. lobata: Low pH vs. Background pH	5.4225	-23.2679	3.6292	-6.4113	2.39E-09	12788188a782540b00a08e41ecb8fb02	*Tistlia*
P. lobata: Low pH vs. Background pH	5.9591	-23.3778	3.6290	-6.4419	2.06E-09	6494b66fa5ee1df4372d0d8c3c231f27	*Prosthecochloris*
P. lobata: Low pH vs. Background pH	5.8751	-23.4124	3.6290	-6.4514	2.04E-09	72ae658499d1d3fe3774d635a2590579	SAR202 clade
P. lobata: Low pH vs. Background pH	119.5865	-23.7003	3.5991	-6.5850	8.85E-10	e447302ae83d41b671bb0a4723dd1997	*Prosthecochloris*
P. lobata: Low pH vs. Background pH	9.8462	-23.9579	3.6284	-6.6029	8.34E-10	73ad979bab097bc66c722f16f922f720	unclassified Caldilineaceae
P. lobata: Low pH vs. Background pH	25.2143	-24.1033	3.6278	-6.6440	7.22E-10	5f8e501dfbd47255253b499e791e3fee	*Aquimarina*
P. lobata: Low pH vs. Background pH	10.7794	-24.2763	3.6283	-6.6908	6.12E-10	451e4d86a83b7f8787a735eeac57b1be	SAR202 clade
P. lobata: Low pH vs. Background pH	18.1246	-24.8571	3.6280	-6.8515	3.02E-10	b9db25ef74e6b79bfd9a62aa92a29893	SAR202 clade
P. lobata: Low pH vs. Background pH	25.7361	-25.4163	3.6278	-7.0060	1.16E-10	c33b899214e1808739e46f1b36f6d08f	SAR202 clade
P. lobata: Low pH vs. Background pH	27.1514	-25.4631	3.6278	-7.0189	1.16E-10	2da178c61f200a6d0f73c99dd1ad3197	SAR202 clade

P. lobata: Low pH vs. Background pH	27.0626	-25.5312	3.6278	-7.0377	1.16E-10	433a602c84c043c23596257e61ded7ac	*Aquimarina*
P. lobata: Low pH vs. Background pH	36.5002	-25.8498	3.6277	-7.1257	1.14E-10	19f33434f62e0afaffd1e71ddd99999a	SAR202 clade
P. lobata: Low pH vs. Background pH	44.8439	-26.1634	3.6277	-7.2122	9.11E-11	93ea314b2d01c414170bdf0f53e2b8d9	*Nitrospira*
P. lobata: Mid pH vs. Background pH	33.5972	30.0000	3.5649	8.4155	7.03E-14	edbf61ceeed7720011cfb57d86d29726	Candidatus *Amoebophilus*
P. lobata: Mid pH vs. Background pH	35.2136	25.5910	3.6344	7.0414	1.27E-09	ab05d619e303c592cba7915a716b0d80	unclassified Rhizobiaceae
P. lobata: Mid pH vs. Background pH	62.2584	25.1843	3.6341	6.9299	1.74E-09	268498939751d83deb01f994c20183f2	unclassified Kiloniellaceae
P. lobata: Mid pH vs. Background pH	134.2904	25.0873	3.6340	6.9036	1.74E-09	b059a6d42b6c88bab91bdd9413c8bc341	Candidatus *Amoebophilus*
P. lobata: Mid pH vs. Background pH	55.3525	24.9538	3.6342	6.8664	1.74E-09	acd34548aa8059a78cea573fb923fffe	unclassified Kiloniellaceae
P. lobata: Mid pH vs. Background pH	37.8266	24.9392	3.6343	6.8621	1.74E-09	8b7d5fa6cd4c01e8ade118a5bd5049e9	unclassified Desulfocapsaceae
P. lobata: Mid pH vs. Background pH	30.3809	24.4901	3.6344	6.7383	3.07E-09	5ba318fbe80492d679c5dfcbdd1accf6	unclassified Desulfocapsaceae
P. lobata: Mid pH vs. Background pH	72.9649	24.4544	3.6341	6.7291	3.07E-09	95edabdf27b0c79327ace1b9ecce66091	*Tistlia*
P. lobata: Mid pH vs. Background pH	28.8303	24.3470	3.6345	6.6989	3.30E-09	d72003c4870959dab3950dc4fe450f54	*Woeseia*
P. lobata: Mid pH vs. Background pH	28.8105	24.3025	3.6345	6.6866	3.30E-09	3e36604833e815063f6d0efd35830ef3	unclassified Kiloniellaceae
P. lobata: Mid pH vs. Background pH	23.7774	24.1921	3.6346	6.6560	3.61E-09	fd38cb4468e2c45f4c73a2d1f7c5d5e1	unclassified Kiloniellaceae
P. lobata: Mid pH vs. Background pH	46.9970	23.9887	3.6342	6.6008	4.70E-09	c2433508e8802e7ea43ef214fc813f62	*Tistlia*
P. lobata: Mid pH vs. Background pH	26.1283	23.9792	3.6346	6.5976	4.70E-09	50d59728803137edcdf7e5bc7c00142b	IS-44 clade
P. lobata: Mid pH vs. Background pH	33.0470	23.7945	3.6344	6.5470	5.16E-09	229657d69e3fe30bc94a46697e5616cd1	SAR324_clade
P. lobata: Mid pH vs. Background pH	26.1758	23.7891	3.6346	6.5453	5.16E-09	d42aa4dd9b6f55973db65687260d08e5	MBAE14 clade
P. lobata: Mid pH vs. Background pH	20.2223	23.7665	3.6348	6.5387	5.16E-09	23fc8e630361d724bba3577393fe7d4a	unclassified Nitrosococcaceae
P. lobata: Mid pH vs. Background pH	51.1636	23.6983	3.3728	7.0264	1.27E-09	61124b9645d64cfef82a94e2b9bcf1c01	unclassified Cyclobacteriaceae
P. lobata: Mid pH vs. Background pH	19.5261	23.5811	3.6348	6.4876	6.53E-09	d56be2fe84226d0fff7b8bc502d5d31a	Babeliales
P. lobata: Mid pH vs. Background pH	22.3781	23.4934	3.6347	6.4637	7.35E-09	930218db41fbce6afaddf903e1ae58fe1	*Woeseia*
P. lobata: Mid pH vs. Background pH	20.2632	23.4614	3.6348	6.4547	7.50E-09	5229e9cffbf1ebc862c6027c4f73e969	SAR202 clade
P. lobata: Mid pH vs. Background pH	21.2647	23.2758	3.6347	6.4037	9.02E-09	16e3c6c8b821a237d9581682ba26fcd3	SAR202 clade
P. lobata: Mid pH vs. Background pH	15.4974	23.2636	3.6351	6.3998	9.02E-09	1bf7d17485f086bc0d1a7a4b9e96db7f	Nitrospina
P. lobata: Mid pH vs. Background pH	15.1815	23.2084	3.6351	6.3845	9.51E-09	cc9c1982b0de0412ec1c61df8fce0894	Candidatus *Amoebophilus*
P. lobata: Mid pH vs. Background pH	18.9166	23.1906	3.6348	6.3801	9.51E-09	7dcd0d9e009fa95048f75bb1b12b98121	PS1 clade
P. lobata: Mid pH vs. Background pH	30.4773	23.1790	3.6344	6.3776	9.51E-09	ed914d7621c19d2468decd160bc368731	unclassified Kiloniellaceae
P. lobata: Mid pH vs. Background pH	17.0871	23.1402	3.6349	6.3660	9.96E-09	68f0f52ff99f9d6dcf933b983f63ebba	unclassified Parvibaculaceae
P. lobata: Mid pH vs. Background pH	13.2219	23.1141	3.6353	6.3583	1.00E-08	14921254e2d2c3f24953d31adf2f68b1	BD2-7 clade
P. lobata: Mid pH vs. Background pH	24.8058	23.0604	3.6347	6.3445	1.02E-08	98451532b5cdf21fb94465d7b6f4fa351	*Woeseia*

P. lobata: Mid pH vs. Background pH	14.1423	23.0534	3.6352	6.3418	1.02E-08	89f8e26357e112857a8fc3cf0c96b389	BD2-7 clade
P. lobata: Mid pH vs. Background pH	12.6433	23.0360	3.6353	6.3367	1.03E-08	8ff21b3e49f84dc5af3593bd18a6812e	unclassified Phycisphaeraceae
P. lobata: Mid pH vs. Background pH	11.2226	22.8783	3.6355	6.2930	1.33E-08	39c0ed3d34a1618c2f8f1c1fbeadf87e	unclassified Nitrosomonadaceae
P. lobata: Mid pH vs. Background pH	10.5848	22.8340	3.6356	6.2806	1.38E-08	3ecb7762dcb6f7299b93b7e4cb9c478c	Magnetospira
P. lobata: Mid pH vs. Background pH	12.0292	22.8111	3.6354	6.2747	1.40E-08	b43282e9a80d6887b751870bd8764c20	Nitrospira
P. lobata: Mid pH vs. Background pH	18.3603	22.6856	3.6349	6.2411	1.66E-08	0e355df990c6370d2786d5391d3771dc	Kordiimonas
P. lobata: Mid pH vs. Background pH	10.2524	22.6274	3.6357	6.2237	1.78E-08	67c2b07cfdfa6c17221bdfb8a7b4dbc8	unclassified Kiloniellaceae
P. lobata: Mid pH vs. Background pH	9.6281	22.6161	3.6358	6.2204	1.78E-08	fe84f6ae5382b8ef6769636116343871	unclassified Cyclobacteriaceae
P. lobata: Mid pH vs. Background pH	9.3706	22.5732	3.6359	6.2085	1.83E-08	4f1fb4d15fe41bcb6a3fe38b2e949eed	unclassified Nitrosomonadaceae
P. lobata: Mid pH vs. Background pH	8.6926	22.4275	3.6360	6.1681	2.26E-08	c6261dbc957c59896e1cf29c2c275711	KI89A clade
P. lobata: Mid pH vs. Background pH	14.8238	21.8568	3.6358	6.0115	5.90E-08	6e0d33c30f0a0700fb9d4488aa2fb1f41	Candidatus *Amoebophilus*
P. lobata: Mid pH vs. Background pH	104.5891	10.9280	3.1687	3.4487	0.01688	408a22a5077320f9cc44a93a98f60404	AqS1
P. lobata: Mid pH vs. Background pH	84.0132	10.6119	3.1624	3.3556	0.0233392	edd2af25351863380dc230d0caf547a2	*Pelagibius*
P. lobata: Mid pH vs. Background pH	5.4225	-21.6254	3.6292	-5.9588	7.75E-08	12788188a782540b00a08e41ecb8fb02	*Tistlia*
P. lobata: Mid pH vs. Background pH	5.9591	-21.6820	3.6290	-5.9746	7.15E-08	6494b66fa5ee1df4372d0d8c3c231f271	*Prosthecochloris*
P. lobata: Mid pH vs. Background pH	5.8751	-21.7177	3.6290	-5.9844	6.85E-08	72ae658499d1d3fe3774d635a25905791	SAR202 clade
P. lobata: Mid pH vs. Background pH	25.2143	-22.5011	3.6278	-6.2024	1.85E-08	5f8e501dfbd47255253b499e791e3fee1	*Aquimarina*
P. lobata: Mid pH vs. Background pH	18.1246	-22.5179	3.6280	-6.2068	1.83E-08	b9db25ef74e6b79bfd9a62aa92a298931	SAR202 clade
P. lobata: Mid pH vs. Background pH	10.7794	-22.5329	3.6283	-6.2103	1.83E-08	451e4d86a83b7f8787a735eeac57b1be1	SAR202 clade
P. lobata: Mid pH vs. Background pH	9.8462	-22.5711	3.6284	-6.2207	1.78E-08	73ad979bab097bc66c722f16f922f7201	unclassified Caldilineaceae
P. lobata: Mid pH vs. Background pH	27.0626	-23.6388	3.6278	-6.5160	5.64E-09	433a602c84c043c23596257e61ded7ac1	*Aquimarina*
P. lobata: Mid pH vs. Background pH	25.7361	-23.7116	3.6278	-6.5361	5.16E-09	c33b899214e1808739e46f1b36f6d08f1	SAR202 clade
P. lobata: Mid pH vs. Background pH	27.1514	-23.7962	3.6278	-6.5594	5.16E-09	2da178c61f200a6d0f73c99dd1ad31971	SAR202 clade
P. lobata: Mid pH vs. Background pH	36.5002	-24.2345	3.6277	-6.6804	3.30E-09	19f33434f62e0afaffd1e71ddd99999a1	SAR202 clade
P. lobata: Mid pH vs. Background pH	44.8439	-24.4802	3.6277	-6.7482	3.07E-09	93ea314b2d01c414170bdf0f53e2b8d91	*Nitrospira*
P. rus: Low pH vs. Background pH	78.0628	25.1996	3.5340	7.1306	4.67E-11	05633648b6fb92157a640769eb5ace44	*Endozoicomonas*
P. rus: Low pH vs. Background pH	107.1260	24.4563	3.3825	7.2304	3.00E-11	4f932acf10e1f45da77a6e889c706429	*Endozoicomonas*
P. rus: Low pH vs. Background pH	26.2736	-23.5560	3.1810	-7.4052	1.33E-11	aad780685c770f7283cd392f70376023	unclassified Cellvibrionaceae
P. rus: Low pH vs. Background pH	110.9006	-26.1296	3.5339	-7.3940	1.33E-11	5ef6eb8dac9322978245be6f8043bc35	*Algicola*

Supplemental Table S3. Statistical comparisons of log-transformed trace element concentrations in coral skeleton across sites. Sites (within a species) were compared by the 1-way ANOVA with bonferroni adjustments. Bold = significantly different across sites at p ≤ 0.002.

Coral Species		Trace Elements																				
		Li	B	Al	P	V	Mn	Co	Ni	Cu	Zn	Mo	Cd	Sb	Ba	Nd	Pb	U	Na	Mg	Fe	Sr
Pocillopora eydouxi	F value	2.37	0.25	0.77	1.58	3.24	**254.71**	0.06	1.17	0.14	0.23	2.57	7.72	0.89	7.04	4.35	3.82	0.38	1.05	1.95	1.34	0.64
	p value	0.133	0.786	0.485	0.245	0.075	**0.0001**	0.940	0.350	0.873	0.795	0.117	0.007	0.435	0.011	0.038	0.052	0.691	0.383	0.184	0.302	0.547
Porites lobata	F value	4.50	9.30	0.61	0.01	5.63	**154.12**	1.48	0.23	0.68	8.60	0.09	5.43	1.38	3.74	9.50	0.58	4.97	10.00	3.81	**26.69**	6.85
	p value	0.035	0.004	0.557	0.987	0.021	**0.0001**	0.266	0.797	0.542	0.005	0.919	0.021	0.289	0.055	0.004	0.576	0.027	0.003	0.058	**0.0001**	0.010
Porites rus	F value	0.47	3.38	0.69	ND*	2.43	**52.33**	3.78	0.01	1.01	4.47	0.03	2.99	2.81	0.25	2.26	2.23	0.04	1.80	4.91	4.13	0.44
	p value	0.637	0.068	0.542		0.130	**0.0001**	0.053	0.988	0.394	0.038	0.970	0.088	0.103	0.780	0.147	0.150	0.962	0.207	0.030	0.043	0.652

*values measures were less than Limit of Detection (LOD)

Supplemental Table S4. Results of Canonical Correspondence Analysis of each coral species in relation to vent associated predictor variables.

Coral Species		Axis 1	Axis 2	p (Monte-Carlo Permutation test)
Pocillopora eydouxi	a. Summary statistics			
	Eigenvalues	0.01	0.004	0.046
	Cumulative % variation explained	91.9	5.8	
	b. Correlation coefficients of predictor			
	Seawater pH	-0.67	0.34	
	Manganese	0.74	-0.21	
	Iron	0.05	0.63	
	Aluminum	0.23	0.03	
Porites lobata	a. Summary statistics			
	Eigenvalues	0.68	0.12	0.006
	Cumulative % variation explained	79.03	14.22	
	b. Correlation coefficients of predictor			
	Seawater pH	-0.89	-0.08	
	Manganese	0.92	0.04	
	Iron	0.81	0.05	
	Aluminum	-0.28	0.59	
Porites rus	a. Summary statistics			
	Eigenvalues	0.13	0.05	0.03
	Cumulative % variation explained	71.0	27.0	
	b. Correlation coefficients of predictor			
	Seawater pH	0.71	-0.27	
	Manganese	-0.63	-0.38	
	Iron	-0.14	-0.51	
	Aluminum	0.24	-0.64	

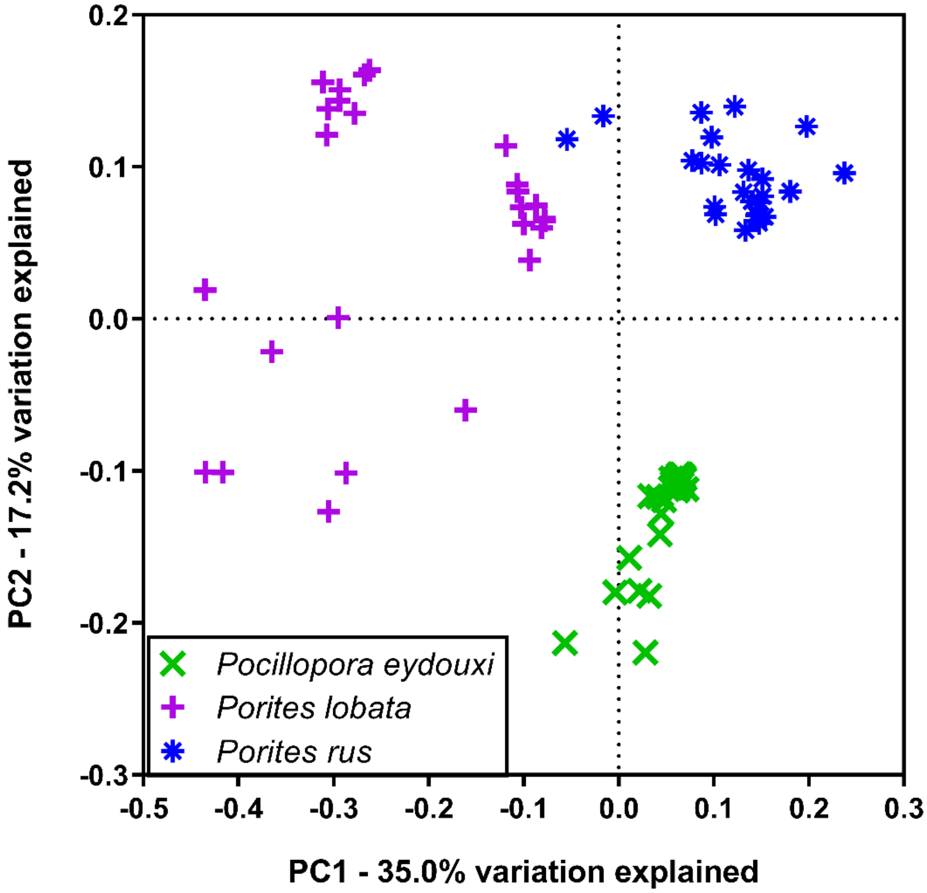

Supplemental Figure S1. Principle Coordinate analysis (PCoA) of the weighted UniFrac distance matrices for all three coral species. Green cross = *Pocillopora eydouxi* samples; purple plus = *Porites lobata* samples; blue star = *Porites rus* samples.

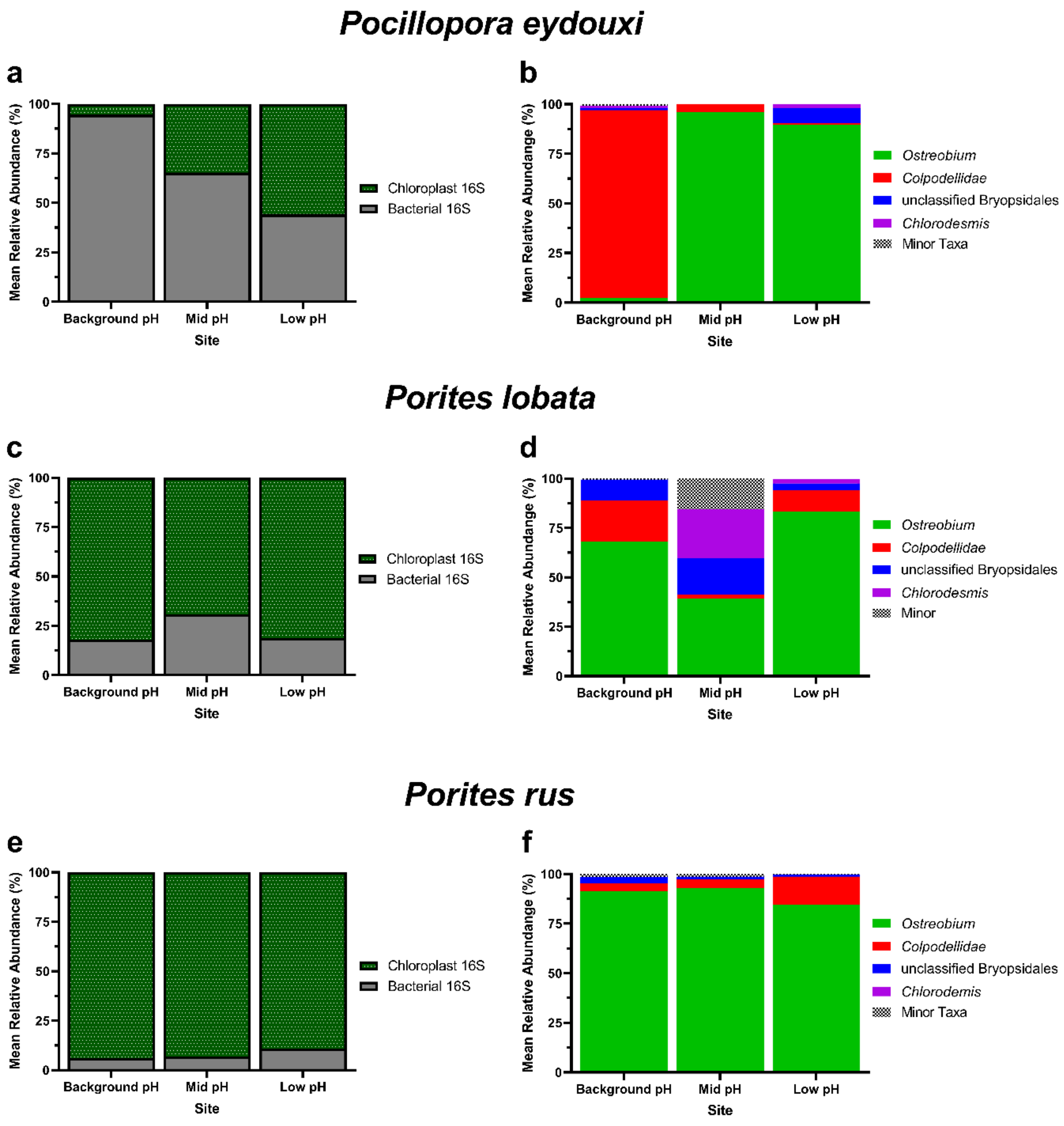

Supplemental Figure S2. Mean percent abundance of chloroplast-derived versus bacterial sequences for a) *Pocillopora eydouxi*, c) *Porites lobata*, and e) *Porites rus* coral samples at Background pH, Mid pH and Low pH sites. Mean percent abundance of chloroplast-derived sequences classified at the phylogenetic level of Genus or Family for b) *Pocillopora eydouxi*, d) *Porites lobata*, and f) *Porites rus* coral samples at Background pH, Mid pH and Low pH sites. Taxonomy of chloroplast-derived reads was assigned using the Protist Ribosomal Reference (PR^2) database. All other taxa which were < 1% of the total community are grouped under category 'Minor Taxa'.

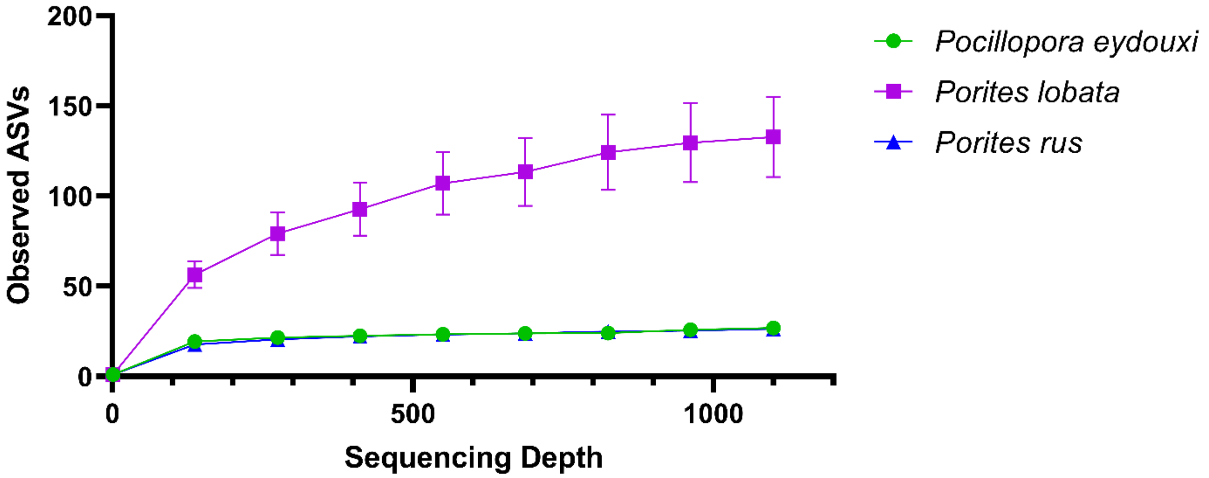

Supplemental Figure S3. Rarefaction curves for all three coral species indicating richness of observed bacterial Amplicon Sequence Variants after filtering out Unassigned, Mitochondrial, and Chloroplast assigned reads as well as rarefying to 1,100 reads per sample. Green circle = *Pocillopora eydouxi*; purple squares = *Porites lobata*; blue triangle = *Porites rus*. Data represent mean ± standard error.
